# Inferences of individual differences in response to tripterysium glycosides across patients with Rheumatoid arthritis using a novel ceRNA regulatory axis

**DOI:** 10.1002/ctm2.185

**Published:** 2020-10-09

**Authors:** Yanqiong Zhang, Xiaoyue Wang, Weijie Li, Hailong Wang, Xiaoli Yin, Funeng Jiang, Xiaohui Su, Wenjia Chen, Taixian Li, Xia Mao, Minqun Guo, Quan Jiang, Na Lin

**Affiliations:** ^1^ Institute of Chinese Materia Medica China Academy of Chinese Medical Sciences Beijing P. R. China; ^2^ Key Laboratory of Beijing for Identification and Safety Evaluation of Chinese Medicine Institute of Chinese Materia Medica China Academy of Chinese Medical Sciences Beijing P. R. China; ^3^ Division of Rheumatology Guang An Men Hospital China Academy of Chinese Medical Science Beijing P. R. China; ^4^ Guangdong Key Laboratory of Clinical Molecular Medicine and Diagnostics South China University of Technology Guangzhou P. R. China; ^5^ College of Life Science South China Normal University Guangzhou P. R. China

**Keywords:** competitive endogenous RNA, personalized medicine, rheumatoid arthritis, tripterysium glycosides

## Abstract

**Background:**

To identify biomarkers for guiding therapy and predicting clinical response of Tripterysium Glycosides Tablets (TGT) treatment is an urgent task due to individual differences in TGT response across rheumatoid arthritis (RA) patients. Competing endogenous RNA (ceRNA) regulatory system may influence drug response with involvement in diverse biological processes. Herein, we aimed to identify a TGT response‐related ceRNA axis.

**Methods:**

A TGT response‐related ceRNA axis was screened according to clinical cohort‐based RNA expression profiling, lncRNA‐mRNA coexpression, and ceRNA network analyses. Its clinical relevance was evaluated by computational modeling. Regulatory mechanisms of ceRNA axis were also experimentally investigated.

**Results:**

The ceRNA regulatory axis combined with lncRNA ENST00000494760, miR‐654‐5p, and C1QC was identified as a candidate biomarker for RA patients' response to TGT. Both ENST00000494760 and C1QC mRNA expression were significantly lower, while miR‐654‐5p expression was dramatically higher in TGT responders than nonresponders. Its clinical relevance was verified by computational modeling based on both independent clinical validation cohort and collagen‐induced arthritis (CIA) mice. Mechanistically, miR‐654‐5p directly bound to the 3′‐untranslated region of both ENST00000494760 and C1QC mRNA to inhibit their expression. Moreover, miR‐654‐5p suppressed C1QC mRNA expression, but ENST00000494760 bound to miR‐654‐5p and relieved its repression on C1QC mRNA, leading to RA aggressive progression and weak TGT response.

**Conclusions:**

LncRNA ENST00000494760 overexpression may sponge miR‐654‐5p to promote C1QC expression in RA patients. This novel ceRNA axis may serve as a biomarker for screening the responsive RA patients to TGT treatment, which will allow improved personalized healthcare.

## Graphical abstract

Molecular mechanism underlying individual differences in response to Tripterysium Glycosides Tablets (TGT) is inadequate and the solution is limited. A ceRNA regulatory axis with lncRNA ENST00000494760, miR‐654‐5p and C1QC was identified as a candidate biomarker for rheumatoid arthritis (RA) patients' response to TGT. ENST00000494760 may sponge miR‐654‐5p to promote C1QC expression, leading to RA aggressive progression and weak TGT response.

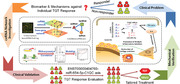

## 1 BACKGROUND

Individual differences in drug response across patients usually exist in the process of treating diseases, and mean that there may be a fraction of patients who are at risk of unnecessary exposure to side‐effects due to ineffective treatment. To overcome this obstacle and to improve the personalized medicine, accumulating studies have been focused on the identification of novel molecular signatures for predicting the drug response of individual patients, which may help to plan treatment. Tripterysium Glycosides Tablets (TGT, Leigongteng DuoganTablets), composed of extracts of a traditional Chinese herb *Tripterygium wilfordii* Hook F (*Tw*HF), have been approved by China Food and Drug Administration to treat Rheumatoid arthritis (RA). Multiple randomized controlled trials demonstrated that TGT may exert more favorable therapeutic effects than several first‐line disease‐modifying antirheumatic drugs (DMARDs).^1^, ^2^, ^3^ However, only 70% of RA patients respond to TGT and achieve clinical improvement. The understanding of molecular mechanism underlying this interindividual variation in drug response is inadequate, and the solution is limited.

As a novel mode of gene expression regulation, the competitive endogenous RNA (ceRNA) regulatory axis comprises RNA transcripts which can bind to microRNAs (miRNAs) with the same miRNA recognition elements (MREs). miRNAs may regulate gene expression negatively by binding to messenger RNA (mRNAs), while long noncoding RNAs (lncRNAs) can promote the expression of targeted mRNAs by sponging miRNAs through MREs.^4^, ^5^ Among them, lncRNAs, a class of RNAs with more than 200 nucleotides in length, has been indicated to act as ceRNAs for miRNAs and regulate the expression of target mRNA of miRNAs. Accumulating studies have indicated that the ceRNA regulatory axis may be implicated into the pathogenesis of diverse diseases such as RA.^6^, ^7^ Moreover, it is also well‐known that the ceRNA regulatory module may influence drug response with the involvement into diverse biological and pathological processes.^8^, ^9^ Further research on the roles of ceRNA regulatory module in RA responsiveness to drugs is needed to establish novel biomarkers as useful clinical tools.

**FIGURE 1:**
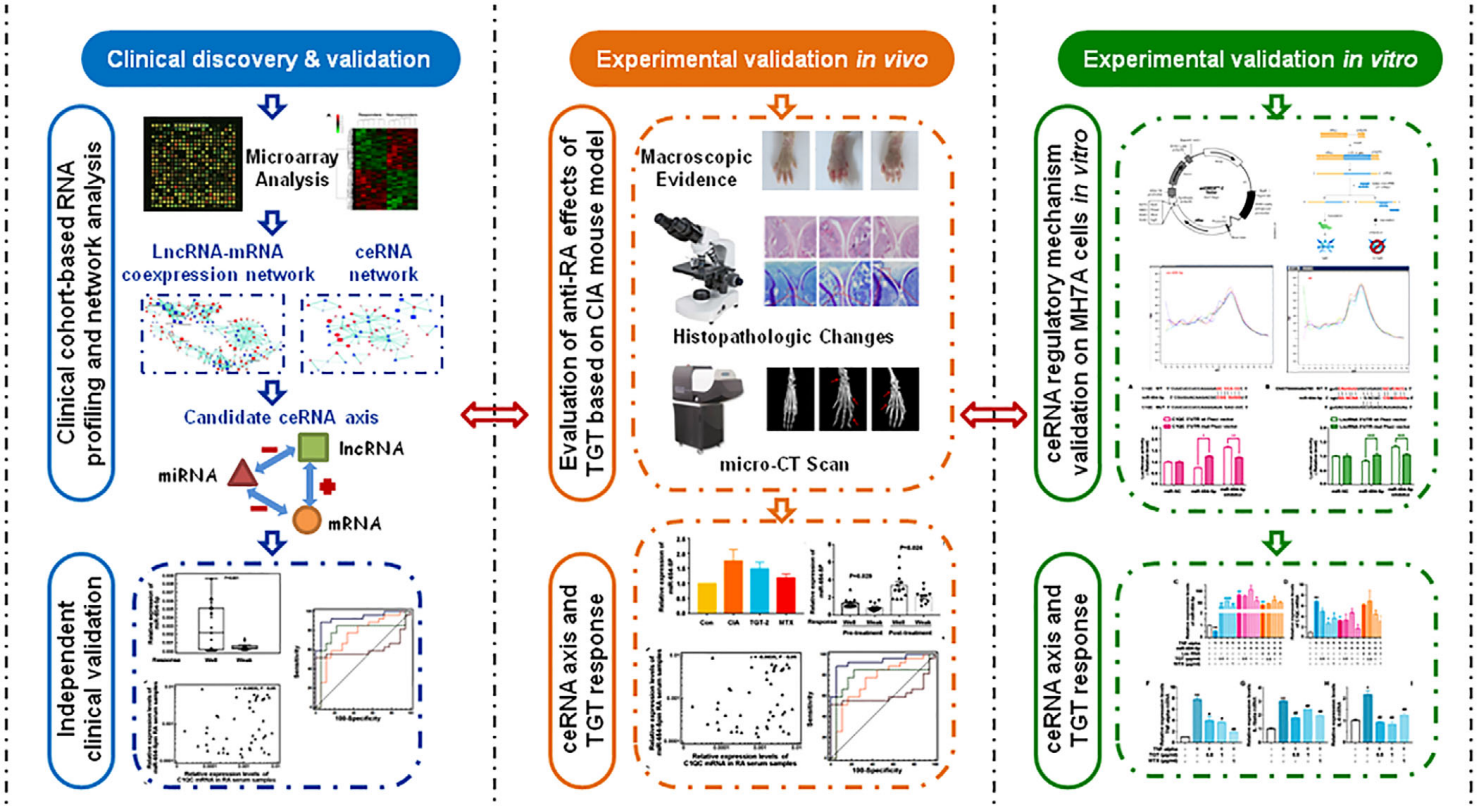
Schematic diagram of our systematic strategies for the identification of a novel ceRNA axis as a biomarker for screening the responsive RA patients to TGT treatment

In our previous studies, we performed the clinical cohort‐based microarray to obtain TGT response‐related mRNA and miRNA expression profiles, and constructed the coexpression network of miRNAs‐target genes and the gene‐gene interaction network to identify four candidate miRNA biomarkers and six candidate gene biomarkers which were predictive of TGT response.^10^, ^11^ Herein, we aimed to identify TGT response‐related ceRNA axes using the expression profiles of miRNAs, mRNAs, and lncRNAs in peripheral blood mononuclear cells (PBMCs) between TGT responders and nonresponders. TGT response‐related lncRNA‐mRNA coexpression network and ceRNA network were constructed and a ceRNA regulatory axis with topological importance was screened. Then, the differential expression patterns of the RNAs in the ceRNA regulatory axis were validated by real‐time quantitative PCR (qPCR) using an independent clinical validation cohort. The clinical relevance of the ceRNA regulatory axis to TGT response was also evaluated by computational modeling based on partial‐least‐squares (PLS) algorithm. After that, the binding efficiency of lncRNA and mRNA to miRNA was validated by luciferase reporter assay using human RA synovial MH7A cells, and the associations of the ceRNA axis with TGT efficacy were further evaluated using the in vitro and in vivo experiments (Figure 1).

## 2 METHODS

### 2.1 Ethics statement

Our clinical sample collection was performed based on the guidelines in the Declaration of Helsinki for humans. Research Ethics Committee of Guang'anmen Hospital approved this study. All patients received the informed and written consent before the sample collection.

Animal experiments in this study were approved by Institute of basic theory for Chinese Medicine, China Academy of Chinese medicine science, China.

### 2.2 Clinical cohorts

Fifty‐one patients with RA, including 14 men and 37 women (age range 25‐84 years old, median age = 58.3 years old), were enrolled from the Department of Rheumatology, Guang'anmen Hospital from January 2015 to October 2019. The inclusion criteria, the treatment of patients, and the definition of responders and nonresponders to TGT were all referred to our previous studies.^10^, ^11^

We divided all 51 patients with RA into a discovery cohort (n = 6, 3 responders and 3 nonresponders) and a validation cohort (n = 45, 27 responders and 18 nonresponders) (Table 1 and Supporting information Table S1). The discovery cohort was used to detect the lncRNA, miRNA, and mRNA expression profiles in PBMCs and to train the parameters in our PLS model for the prediction of TGT response. The validation cohort was used to detect the expression levels of candidate RNA biomarkers via qPCR and to assess the prediction performance of our PLS model.

**TABLE 1:** Clinical and laboratory parameters of RA patients enrolled in the current study

Parameters	Discovery cohort (n = 6)	Validation cohort (n = 45)
Age (years, mean, range)	51.5 (45‐55)	58.9 (25‐84)
Gender (male/female)	2/4	12/33
Erythrocyte sedimentation rate (ESR, mm, mean)	27.8	38.6
C‐reactive protein (CRP, mg/dL, mean)	30.0	19.9
Positive rheumatoid factor (n, %)	4, 66.7	24, 53.3
Positive anticyclic citrullinated peptide (CCP) antibodies (n, %)	4, 66.7	24,53.3

### 2.3 RNA expression profiling and differential expression data analysis

Affymetrix miRNA 4.0 and EG1.0 arrays were respectively used to detect the expression of miRNAs, lncRNAs, and mRNAs in PBMCs obtained from TGT responders and nonresponders. The microarray detection was carried out by Shanghai GMINIX Bio. Co. (Shanghai, China). The microarray data were uploaded to NCBI Gene Expression Omnibus and publicly available under the accession numbers GSE106893 and GSE106894.

The dysregulated miRNAs, lncRNAs, and mRNAs between TGT responder and nonresponder groups were screened by the cutoffpoints of fold change > 1.2 or < 0.83, *P* < .05 and False Discovery Rate (FDR ) > = 1.0 through the RVM *t*‐test and the FDR analysis. The hierarchical clustering analysis was performed using the heat map package in R (version 1.0.2, R Core Team, Vienna, Austria).

### 2.4 Coexpression network of the dysregulated lncRNAs and mRNAs

On the basis of the normalized signal intensity of differentially expressed lncRNAs and mRNAs, R function cor. test (Hmisc and corrplot) was used to compute the coefficient of the Pearson's correlation of lncRNA‐mRNA pairs. The correlation value cutoff value of the significant correlation pairs was 0.99. The coexpression networks of the differentially expressed lncRNAs and mRNAsin TGT‐responder and ‐nonresponder groups were respectively constructed. In each network, the number of lncRNAs or mRNAs correlated to a given mRNA or a given lncRNA is the degree of that mRNA or lncRNA. To measure the centrality of a mRNA or a lncRNA within a network, the relative degree was calculated as the ratio of the degree value of a gene or a lncRNA to the biggest degree in a network. While considering different networks of TGT‐responder and ‐nonresponder groups, core lncRNAs or mRNAs were screened using the relative degree differences between the two groups.

### 2.5 ceRNA regulatory network

ceRNA regulatory network was established to investigate the regulatory mechanisms among the dysregulated miRNAs, lncRNAs, and mRNAs between TGT responder and nonresponder groups. The MRE among RNAs was predicted and the free energy was computed to find the competition relationship among miRNAs, lncRNAs, and mRNAs. At first, miRNA‐mRNA, miRNA‐lncRNA relationships were predicted by TargetScan (Release 7.2, www.targetscan.org) and miRanda (version 3.3a, www.microrna.org). The predictive results both of TargetScan and miRanda were selected for the following analysis. Then, the Pearson correlation coefficients of miRNA‐mRNA and miRNA‐lncRNA pairs were calculated. The correlation value cutoff was 0.99.

### 2.6 Animals

A total of 170 male DBA‐1 mice (7‐9 weeks, weight 19.6 ± 1.5 g) were obtained from Shanghai SLAC Laboratory Animal Co., Ltd (license No.: SCXK 2017‐0007, Shanghai, China), and all maintained in a room with a constant temperature of 24 ± 1°C and with a 12‐h light and dark cycle. The mice were allowed free access to water and food.

### 2.7 Collagen‐induced arthritis (CIA) modeling and grouping

Collagen‐induced arthritis (CIA) mouse model was established according to the protocol described in our previous study.^12^ Briefly, the mice were immunized subcutaneously in the tail with 100 mg of type II collagen (CII, Cat No. #20022, Chondrex, Redmond, USA) emulsified in Complete Freund's Adjuvant (CFA, Cat No. #7001, Chondrex) and boosted 3 weeks later via the same route with CII emulsified in CFA. The mice of normal control group were also subcutaneously injected the saline solution with the same dosage.

For RNA expression and PLS model validation, when arthritis symptoms were appeared, the mice were randomly divided in six groups (5 mice for normal control group and 10 mice per treatment group): normal control (Con), CIA model (CIA), CIA model with the treatment of 13 mg/mL (the clinical daily dosage of patients with RA) of TGT (CIA‐TGT‐1), CIA model with the treatment of 26 mg/mL (two times of the daily dosage for patients with RA in clinics) of TGT (CIA‐TGT‐2), CIA model with the treatment of 52 mg/mL (four times of the daily dosage for patients with RA in clinics) of TGT (CIA‐TGT‐4), and CIA model with the treatment of 3 mg/kg (four times of the daily dosage for patients with RA in clinics) of methotrexate (CIA‐MTX).

For evaluating the regulative correlations in ENST00000494760‐miR‐654‐5p‐C1QC axis and its associations with the response of TGT, the mice were randomly divided in 10 groups (5 mice for normal control and CIA groups, 3 mice for CIA‐TGT and CIA‐MTX groups, and 8 mice per treatment group): normal control (Con), CIA model (CIA), CIA model with the treatment of 26 mg/mL of TGT (CIA‐TGT), CIA model with the treatment of 3 mg/kg of methotrexate (CIA‐MTX), CIA model with the treatment of blank plasmid (CIA‐miR‐NC), CIA model treated with blank plasmid, and 26 mg/mL TGT (CIA‐miR‐NC‐TGT), CIA model treated with miR‐654‐5p plasmid (CIA‐miR‐654‐5p), CIA model treated with miR‐654‐5p plasmid and 26 mg/mL TGT (CIA‐miR‐654‐5p‐TGT), CIA model treated with miR‐654‐5p‐ENST00000494760 plasmid (CIA‐miR‐654‐5p‐lncRNA), CIA model treated with miR‐654‐5p‐ENST00000494760 plasmid and 26 mg/mL TGT (CIA‐miR‐654‐5p‐lncRNA‐TGT).

TGT was administered intragastrically at 13/26/52 mg/kg once a day, while MTX [TonghuaMaoxiang Pharmaceutical Co. Ltd (Lot number:170501, Changchun, China)] was administered intragastrically at 3 mg/kg every 3 days and at 1 day interval. All tablets were dissolved in 0.3% Carboxymethylcellulose sodium salt (CMC‐Na, C6621, Biotopped, Beijing, China).

CIA mice in CIA‐miR‐654‐5pgroup received intraperitoneal injection of 5×10^7^ TU of pLVGFP‐pre‐miR‐654on days 28, 35, 42. CIA mice in CIA‐miR‐NC group were injected with the same doses of LV vectors on days 28, 35, 42. CIA mice in CIA‐miR‐654‐5p‐lncRNAgroup were injected with the same doses of pLVGFP‐pre‐miR‐654andpLVSO2‐ENST00000494760 on days 28, 35, 42.

### 2.8 Lentiviral production and transfection

The pLVGFP and PLVSO2 expression constructs containing mouse pre‐miR‐654‐5p and ENST00000494760 (Guangzhou Hui Yuan Pharmaceutical Technology Co. LTD, P. R. China) are LV‐based vectors in which the miR‐654 precursor molecule/ENST00000494760 is cloned downstream of the cytomegalovirus promoter and carries the reported gene GFP. Recombinant LV vectors were produced by cellular transfection with pLVGFP‐pre‐miR‐654‐5p/pLVSO2‐ENST00000494760 or a scrambled construct as a negative control (Guangzhou Hui Yuan Pharmaceutical Technology Co. Ltd), with the packaging plasmid pPACK‐H1‐GAG, pPACK‐HI‐REV, and pVSV‐G. Cellular transfections were performed using the Turbofect (R0531, Thermo scientific). pLVGFP‐miR‐654/pLVSO2‐ENST00000494760 and the corresponding control were harvested and concentrated by ultracentrifugation, and viral titers (in transduction units) were determined by qPCR.

### 2.9 Assessment of the severity of arthritis

The arthritis scores of CIA mice in each paw were measured every 3 days to evaluate the arthritis severity as described previously.^12^ The levels of IgG2a, interleukin‐6 (IL‐6), and tumor necrosis factor‐alpha (TNF‐α) in mice' sera were examined by the corresponding Enzyme‐linked immunosorbent assay (ELISA) kits (IgG 2a, ab133046, Abcam, Cambridge, UK; IL‐6, EK282HS, Multisciences, Hangzhou, P. R. China; TNF‐α, EK206/3, Multisciences) using Multiskan MK3 enzyme‐labeled instrument.

### 2.10 Histopathologic assessment and microcomputed tomography (micro‐CT)

The knee joints obtained from different groups were dissected, fixed in 10% neutral buffered formalin immediately, decalcified by 10% nitric acid for up to 2 days at room temperature, and embedded in paraffin. Tissue sample sections (4 µm) were mounted on common slides for staining with hematoxylin and eosin (H&E) or toluidine blue (TB). Histological observation was performed based on inflammatory cell infiltration, angiogenesis, synovial hyperplasia, and joint damage. All sections were randomized and evaluated by two trained pathologists in a blinded manner.

The left hind paw of mice in different groups were dissected, fixed in 10% neutral buffered formalin for at least 48 h. Then, all specimens were scanned with micro‐CT system (Skyscan1176, Kontich, Belgium). In brief, a voxel size of 9 µm and a rotation step of 0.4 were selected. The time taken to completely scan one joint was about 20 min, and the 900 projected images were reconstructed by NRecon 1.6.8.0 software (Bruker Co., Kontich, Belgium). CTvox 2.2.0.0 software (Bruker Co.) was used to reconstruct the three‐dimensional (3D) images of the paws and knee joints. CTAn 1.21.10.0 (Bruker Co.) software was used to detect and analyze the 3D parameters of trabecular bone, including tissue mineral density (TMD, g/cm^3^), bone volume fraction (BV/TV, %), and trabecular number (Tb.N, n).

### 2.11 Cell culture and grouping

The human RA synovial MH7A cell line was purchased from the RIKEN biological resource center in Japan, and cultured in RPMI‐1640 medium (sh30809.01B, HyClone, UT, USA) with 10% fetal bovine serum (1027‐106,Gibco, Carlsbad, CA, USA) at 37°C in a 5% CO_2_ humidified atmosphere.

To determine the anti‐inflammatory efficacy of TGT and its regulatory effects on the ceRNA axis, MH7A cells were divided into: (i) Control group: cells without any stimulation and treatment; (ii) Model group: cells with the stimulation of 10 ng/mL TNF‐α for 24 h; (iii) TGT‐treatment group‐1 (TGT‐0.5): the simulated cells were treated with 0.5 µg/mL TGT for 24 h; (iv) TGT‐treatment group‐2 (TGT‐1.0): the simulated cells were treated with 1.0 µg/mL TGT for 24 h; (v) MTX‐treatment group: the simulated cells were treated with 1.0 µg/mL MTX for 24 h. The concentration of DMSO was less than 1% of the solution.

### 2.12 Oligonucleotides, plasmids, and cellular transfection

To construct a plasmid expressing hsa‐miR‐654‐5p and ENST00000494760, the full‐length human hsa‐miR‐654‐5p sequence (NCBI Reference Sequence:NC_000014.9) and human ENST00000494760 sequence (NCBI Reference Sequence: NR_135599.2) was synthesized and inserted into the PLV.O vector to generate PLV.O‐miR‐654 and PLV.O‐ENST00000494760 (Guangzhou HYY Medical Science Co. LTD, Guangzhou, China). Lentiviruses were produced in HEK 293T cells (ATCC, Manassas, VA, USA). Selection was performed over 12 days by adding 2 µg/mL puromycin (Sigma‐Aldrich, Alcobendas, Spain) for PLV.O‐miR‐654 and PLV.O‐ENST00000494760.

### 2.13 Dual‐luciferase reporter assay

The 3′‐untranslated region of the *C1QC* gene and the full ENST00000494760 sequence containing the seed sites into hsa‐miR‐654‐5p were amplified from human cDNA via PCR and cloned into the 3′end of the psi‐CHECK2 luciferase vector. Mutated versions of each construct were generated by mutating the sequences of hsa‐miR‐654‐5p seed sites (psi‐CHECK2‐wt or mut).

MH7A cells seeded into 96‐well plates were transfected with wt/mut report gene and hsa‐miR‐654‐5p/NC‐mimic using Lipofectamine 2000 (Invitrogen, Carlsbad, CA, USA). The Dual‐luciferase reporter assay system (Promega, Madison, WI, USA) was used examine the luciferase activity at 48 h post‐transfection. The relative luciferase activity was normalized to the Renilla luciferase internal control.

### 2.14 Quantitative PCR analysis

Total RNA was isolated with TRIzol reagent (Cat No. 155596026, Invitrogen), and reverse transcription was used transcriptor First Stander cDNA Synthesis Kit (k1622, Invitrogen). LncRNA or mRNA expression levels in different groups were quantified by using ABI HT7900 real‐time PCR system and UltraSYBR Mixture (CW2602, CoWin Bio., Jiangsu, China). Alternatively, RNA was extracted as above described and used Mir‐X™ miRNA First‐Strand Synthesis Kit (638315, TaKara,Kusatsu, Japan) for reverse transcription reactions. miRNA expression levels in different groups were quantified by using ABI HT7900 real‐time PCR system and TB Green Premix (RR820A, TaKara). U6 was used as internal controls for miRNA, both 18S and GAPDH were used as internal controls form RNAs and lncRNAs. The relative expression of miRNAs, mRNAs, and lncRNAs were calculated by the comparative cycle threshold (CT) method. Supporting information Table S2 listed the primer sequences.

### 2.15 Construction and performance evaluation of TGT‐response prediction model

Following the verification of the associations of the ceRNA axis with TGT response, PLS models for predicting the RA patients' response to TGT and CIA mice were constructed using the RNA expression data of the ceRNA axis in peripheral blood as described previously.^11^, ^13^ The independent dataset test was performed to evaluate the performance of our TGT‐response prediction model based on the average accuracy and area‐under‐curve (AUC) of the receiver‐operating‐characteristic (ROC) curves calculated as our description in the previous studies.^11^, ^13^

### 2.16 Statistical analyses

The data are showed as the mean ± SEM. The differences between the two groups were evaluated by GraphPad Prism version 7.00 (GraphPad Software, La Jolla, CA, USA). Two‐tailed Pearson's correlation analysis was performed to evaluate the correlation of the two variables.“*P* < .05″ refers to the difference with statistical significance.

## 3 RESULTS

### 3.1 ENST00000494760‐miR‐654‐5p‐C1QC axis may be a candidate biomarker for RA patients' response to TGT

Differential expression data analysis screened a total of 1,140 dysregulated lncRNAs (613 upregulated and 527 downregulated), 24 dysregulated miRNAs (7 upregulated and 17 downregulated), and 124 dysregulated genes (60 upregulated and 64 downregulated) in PBMC between the TGT responder and nonresponder groups (Supporting information Figure S1).

**FIGURE 2:**
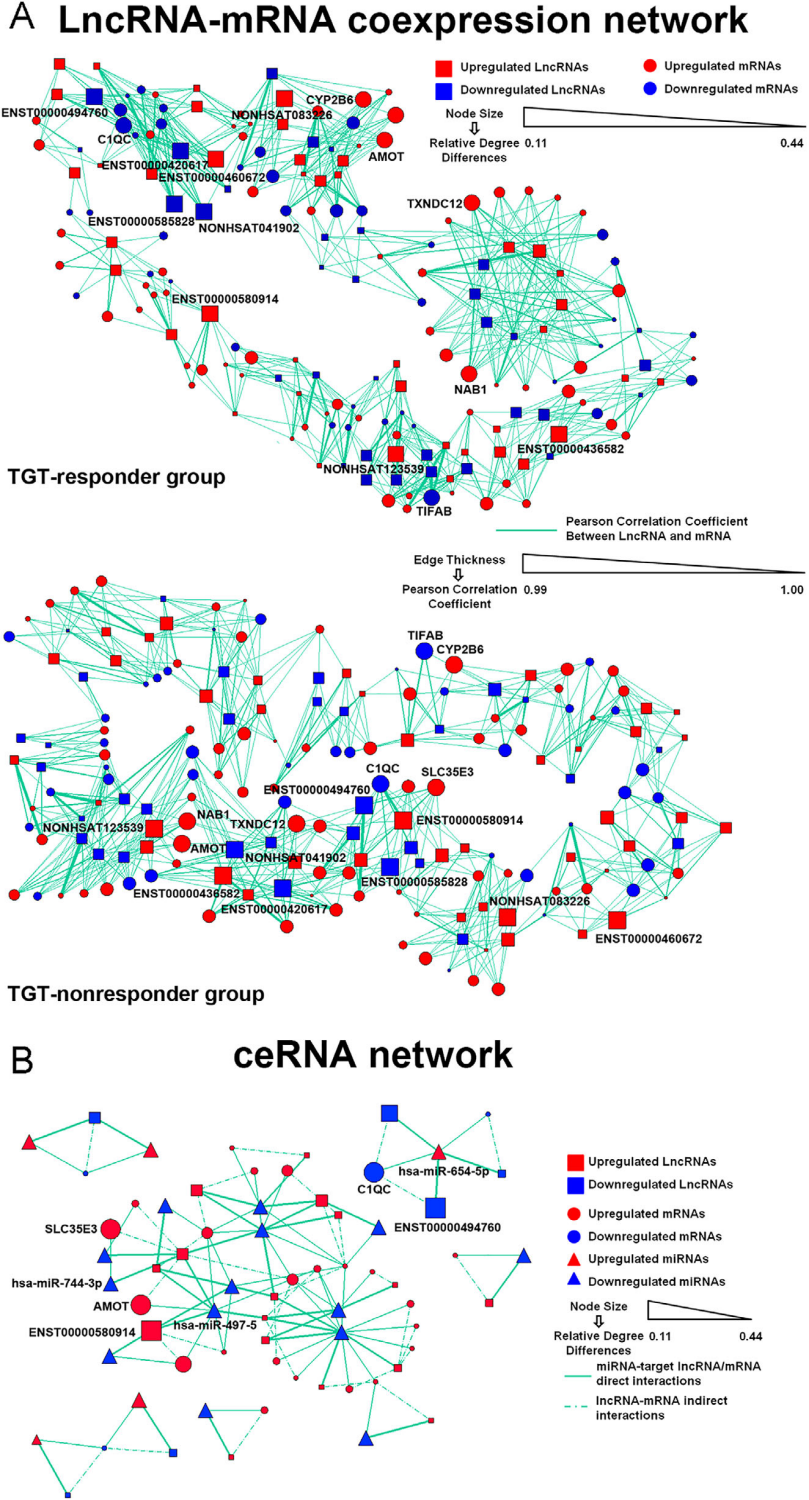
LncRNA‐mRNA coexpression network and ceRNA network associated with patients' response to TGT. (A) LncRNA‐mRNA coexpression network constructed based on Pearson's correlation coefficient of lncRNA‐mRNA pairs. The degree was calculated to measure a mRNA or lncRNA centrality within a network. While considering TGT‐responder and TGT‐nonresponder networks, core lncRNAs, or genes were determined by the degree differences between the two groups. (B) ceRNA network constructed to visualize the ceRNA mechanism based on the differentially expressed lncRNAs, miRNAs, and mRNAs between TGT‐responder and ‐nonresponder groups. In a ceRNA axis, the thicker edge of the miRNA‐target lncRNA pair than that of the miRNA‐target mRNA pair refer to the higher alignment score and thermal stability of the free energy of the target lncRNA to a miRNA than that of the target mRNA to this miRNA

Then, the lncRNA‐mRNA coexpression networks of TGT‐responder and nonresponder groups were constructed based on their expression correlations in the two groups. According to the relative degree differences between the two groups, NONHSAT041902, ENST00000420617, NONHSAT123539, ENST00000436582, and ENST00000494760, as well as CYP2B6, NAB1, TXNDC12, C1QC, and TIFAB were indicated as the top five core lncRNAs or mRNAs (Figure 2A and Supporting information Table S3). In addition, a total of 46 candidate ceRNA axes were identified based on the following criterion: (1) Both mRNA and lncRNA were targeted by the same miRNA; (2) the expression of both mRNA and lncRNA were negatively with this miRNA; (3) the alignment score and the thermal stability of the free energy of the target lncRNA to a miRNA were both higher than that of a target mRNA to this miRNA (Figure 2B and Supporting information Table S4). Among them, only the ENST00000494760‐miR‐654‐5p‐C1QC axis contained the core lncRNA (ENST00000494760) and mRNA (C1QC) with the high relative degree differences between the TGT‐responder and nonresponder groups, thus, this ceRNA axis was selected to be a candidate biomarker for RA patients' response to TGT.

### 3.2 Clinical validation of the differential expression patterns and the predictive efficiency of the ENST00000494760‐miR‐654‐5p‐C1QC axis in TGT response

**FIGURE 3:**
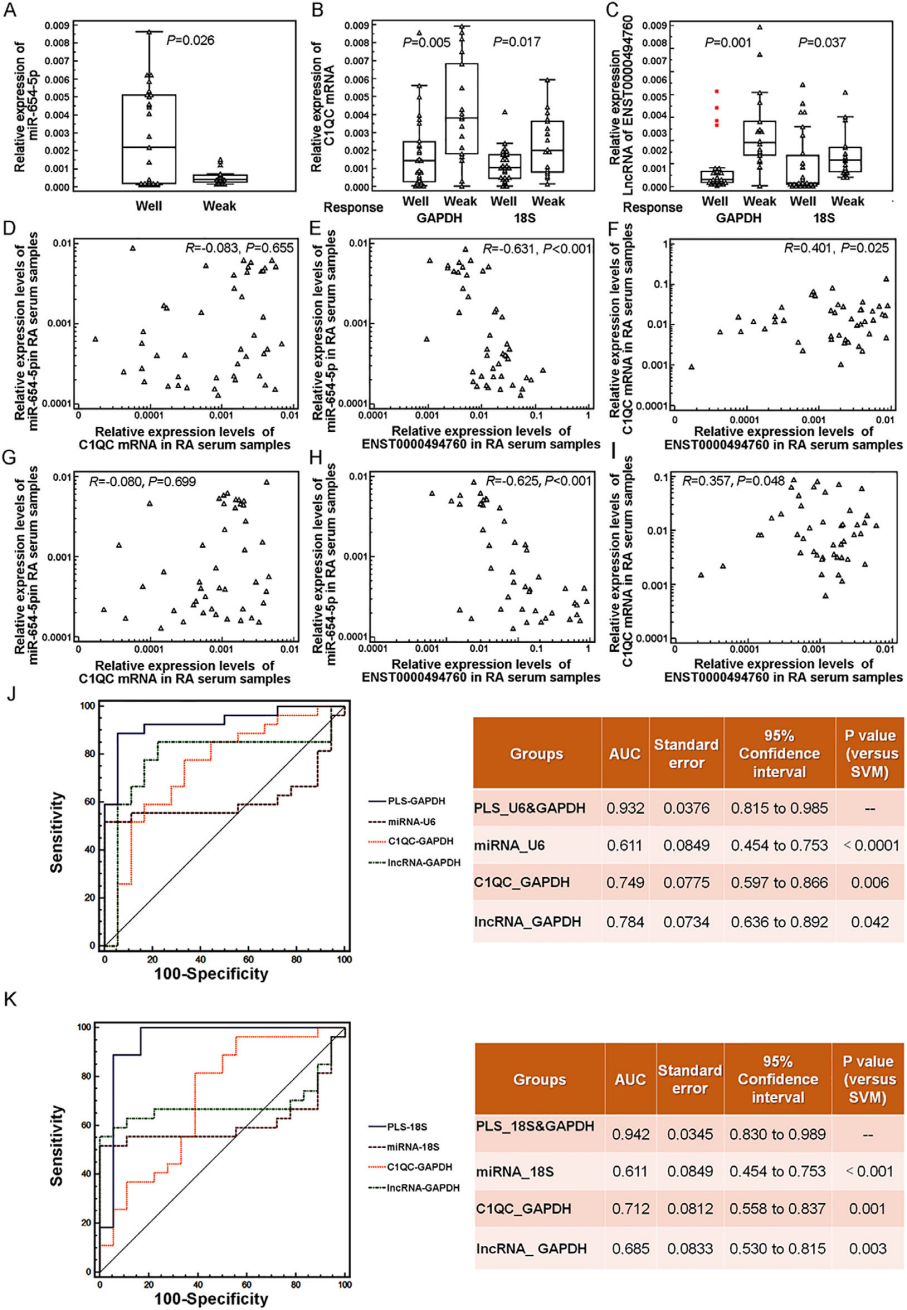
Clinical validation of the differential expression patterns and the predictive efficiency of the ENST00000494760‐miR‐654‐5p‐C1QC axis in TGT response. (A‐C) Serum levels of miR‐654‐5p, C1QC mRNA, and ENST00000494760 in TGT‐responder (well) and ‐nonresponder (weak) groups of the independent validation cohort detected by quantitative PCR analysis, respectively. (D‐I) The correlation graphs of miR‐654‐5p‐C1QC mRNA (GAPDH as internal control), miR‐654‐5p‐ENST00000494760 (GAPDH as internal control), C1QC mRNA‐ENST00000494760 (GAPDH as internal control), miR‐654‐5p‐C1QC mRNA (18S as internal control), miR‐654‐5p‐ENST00000494760 (18S as internal control), and C1QC mRNA‐ENST00000494760 (18S as internal control) pairs.(J, K) ROC comparison of the PLS model which integrated the three RNAs and the single RNA biomarker in predicting patients' response to TGT. GAPDH and 18S were used as internal controls for the detection of CIQC mRNA and ENST00000494760 expression, while U6 was used as an internal control for the detection of miR‐654‐5p expression

The independent validation cohort including 45 patients with RA (27 responders and 18 nonresponders to TGT) was used to verify our microarray data by quantitative PCR analysis. Compared with the TGT‐nonresponder group, the serum levels of miR‐654‐5p were markedly increased in TGT‐responder group (well vs. weak: *P *= .026, Figure 3A‐C), while both C1QC mRNA and ENST00000494760 expression levels in TGT‐responder group were dramatically decreased compared to those in TGT‐nonresponder group [For C1QC mRNA, well vs. weak (GAPDH as internal control): *P *= .005; well vs. weak (18S as internal control): *P *= .017. For ENST00000494760, well vs. weak (GAPDH as internal control): *P *= .001; well vs. weak (18S as internal control): *P *= .037]. In addition, the correlation analysis showed that both C1QC mRNA and ENST00000494760 were positively coexpressed in the validation cohort and negatively with miR‐654‐5p expression (Figure 3D‐I). Notably, the negative correlation between ENST00000494760 and miR‐654‐5p expression was more significant than that between C1QC mRNA and miR‐654‐5p expression (Figure 3D‐I).

To further evaluate the association of the ENST00000494760‐miR‐654‐5p‐C1QC axis to RA patients' response to TGT, we constructed a PLS model using the expression data of the three RNAs in patients' sera of the discovery cohort and assessed the prediction performance of this model using the independent validation cohort. The AUC value and the predictive accuracy of the PLS model using the RNA expression data normalized by GAPDH were 1.000 and 90.50%, respectively, in line with that of the PLS model with 18S as an internal control (the AUC value was 0.888and the accuracy was 92.50%). The ROC comparison was performed to determine the necessity and effectiveness of the PLS model for TGT response. As shown in Figure 3J and K, the AUC values of the PLS model which were integrated miR‐654‐5p, C1QC mRNA, and ENST00000494760 expression data using both GAPDH and 18S were markedly higher than that of the single RNA biomarker (all *P *< .05).

### 3.3 Experimental validation of the differential expression patterns and the predictive efficiency of the ENST00000494760‐miR‐654‐5p‐C1QC axis in TGT response based on CIA mice

#### 3.3.1 TGT treatment ameliorates the arthritis severity of CIA mice

**FIGURE 4:**
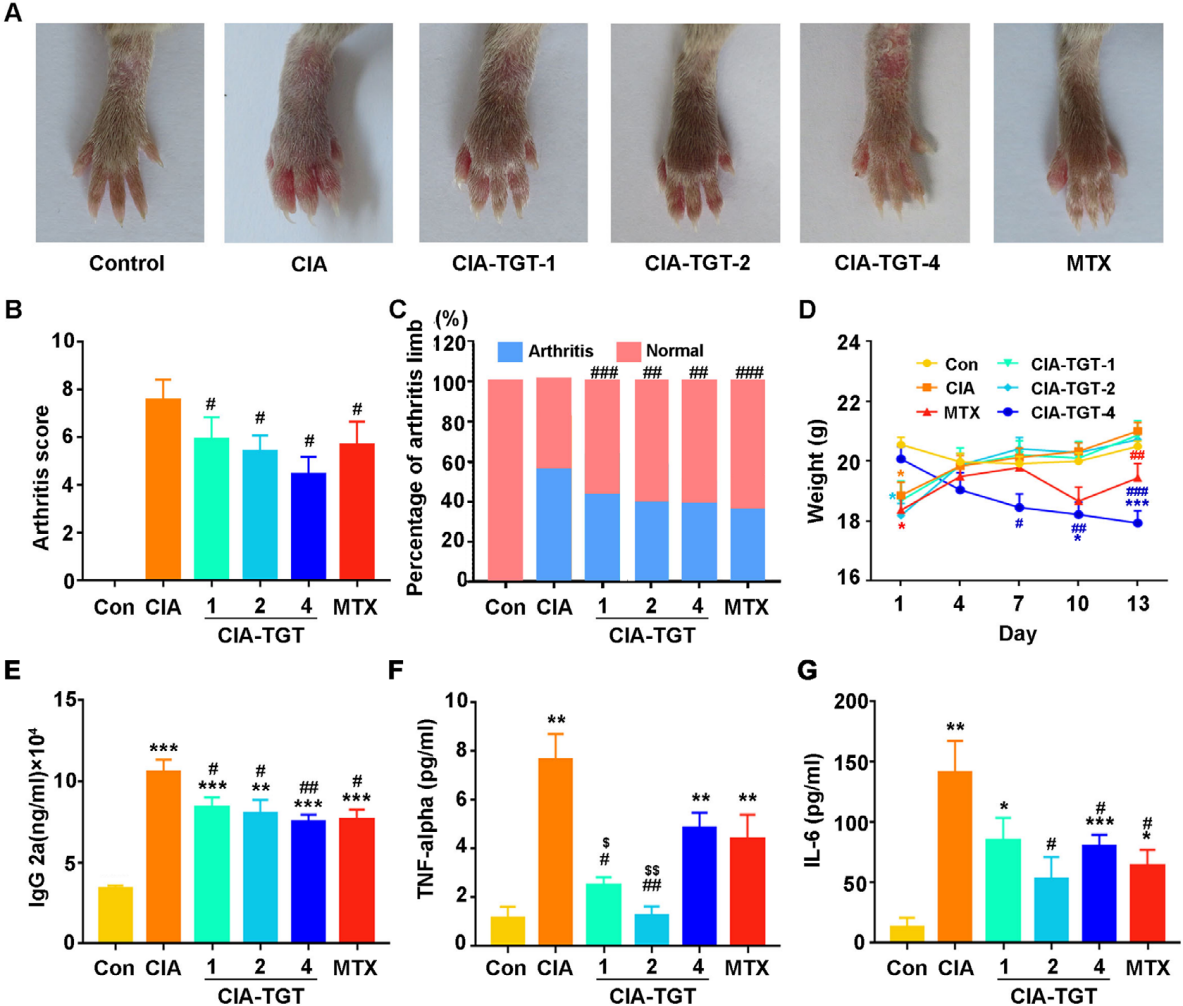
TGT treatment ameliorates the severity of arthritis in CIA mice. (A) Macroscopic evidence of arthritis. (B) Arthritis score. (C) The percentage of the arthritis limbs. (D) Weight. (E) IgG2a. (F) TNF‐α. (G) IL‐6. Data are expressed as mean ± SEM (n = 5‐10);^*^ *P*  < .05, ^**^ *P  *< .01,^***^ *P*  < .001 compared with the normal control group. ^#^ *P * < .05, ^##^ *P * < .01, ^###^ *P * < .001 compared with the CIA model group. ^$^ *P * < .05, ^$$^ *P*  < .01 compared with MTX treatment group

The CIA mouse model was established to evaluate the pharmacological effects of TGT and to investigate the involvement of the ENST00000494760‐miR‐654‐5p‐C1QC axis with the animals' response to TGT. The success rate of CIA mouse model was 85%. Then, the oral administration of TGT with different dosages (CIA‐TGT‐1, ‐2, and ‐4 groups) once per day started from day 1 to 21 following the occurrence of arthritis. The abilities of the CIA mice to feed and function were weakened because of the aggressive redness or swelling in hind limbs. In particular, both the knuckles and ankles were seriously affected by symmetrical arthrocele, which were effectively attenuated by the administration of TGT (Figure 4A). Statistically, TGT dose‐dependently interfered with the increasing arthritis scores and the percentage of arthritis limbs in CIA mice (all *P* < .05, Figure 4B and C). In addition, the marked weight loss was induced by arthritis, which was gradually increased by the treatment of TGT (Figure 4D). However, we observed that the weight values in CIA‐TGT‐4 group continued to loss due to the toxic effects of the large doses of TGT (Figure 4D). Moreover, IgG2a, IL‐6, and TNF‐α levels were dramatically enhanced in CIA mice comparing with the normal mice, while dramatically reduced in both the TGT and MTX treatment groups (all *P *< .05, Figure 4E‐G).

#### 3.3.2 TGT treatment improves the tissue architecture of knees in CIA mice

**FIGURE 5:**
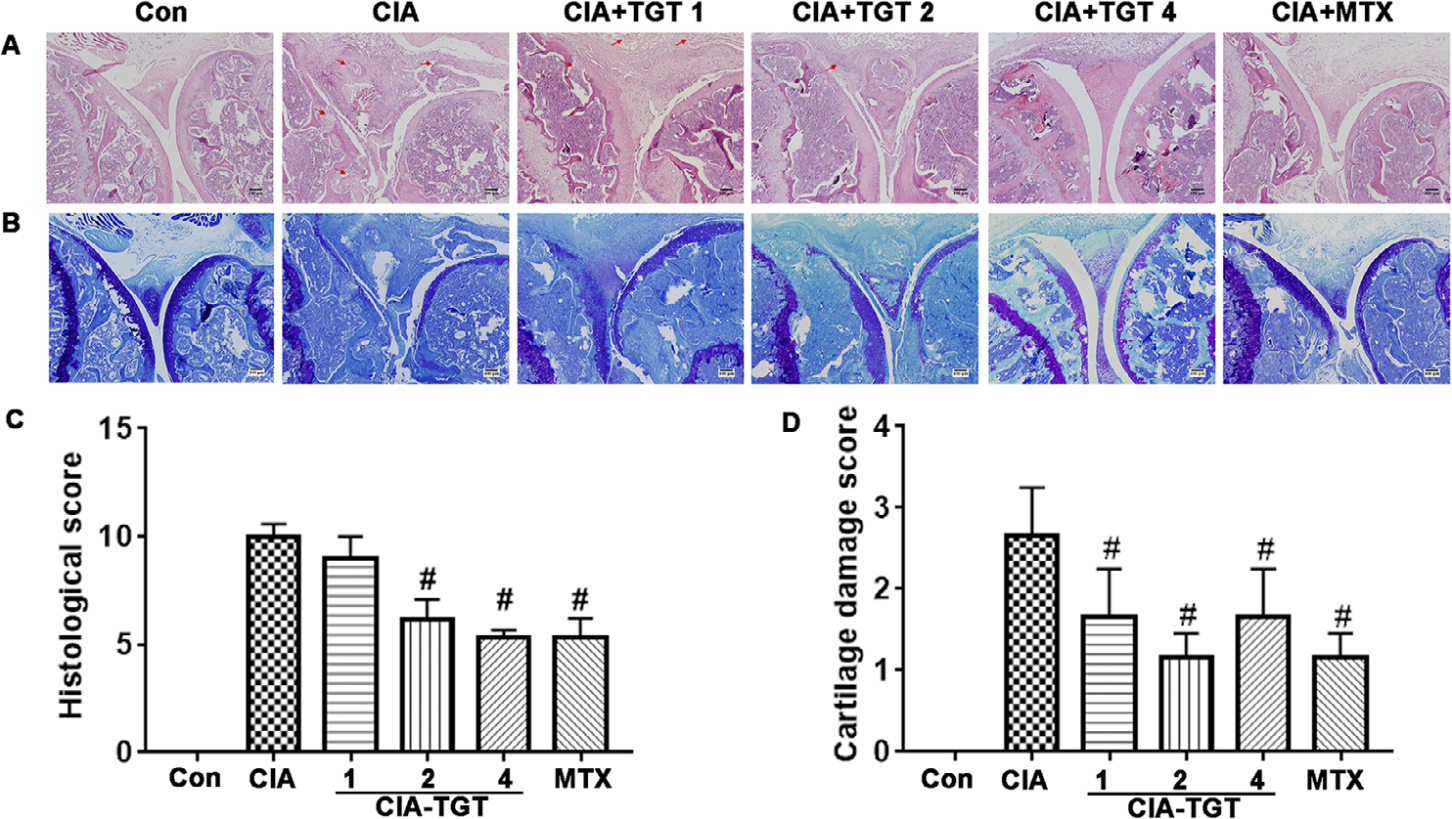
TGT treatment improved the tissue architecture of knees in CIA mice. Representative histopathological images of the knee joint tissues examined by (A) H&E staining and (B) toluidine blue staining. (C) Histological scores and (D) cartilage damage scores were determined by independent observers in a blinded manner (histological scoring was mainly composed of the total scores of inflammatory cell infiltration, synovial hyperplasia, cartilage, and bone destruction and Pannus formation, with each score ranging from 0 to 3. 0, none; 1, mild; 2, moderate; 3, severe. n = 3). ^#^ *P * < .05, compared with the CIA group

Histopathological observations of the knee joint sections of CIA mice showed a distinct infiltration of inflammatory cells, pannus formation, joint destruction, and synovial hyperplasia, which were all significantly attenuated by the treatment of TGT with different dosages (Figure 5A and C). Additionally, toluidine blue staining for cartilage was remarkably decreased in CIA mice compared with normal mice. By contrary, the joints from mice with the treatment of TGT or MXT showed the obvious reduction of all pathological features (Figure 5B and D).

#### 3.3.3 TGT treatment prevents the bone destruction of CIA mice

**FIGURE 6:**
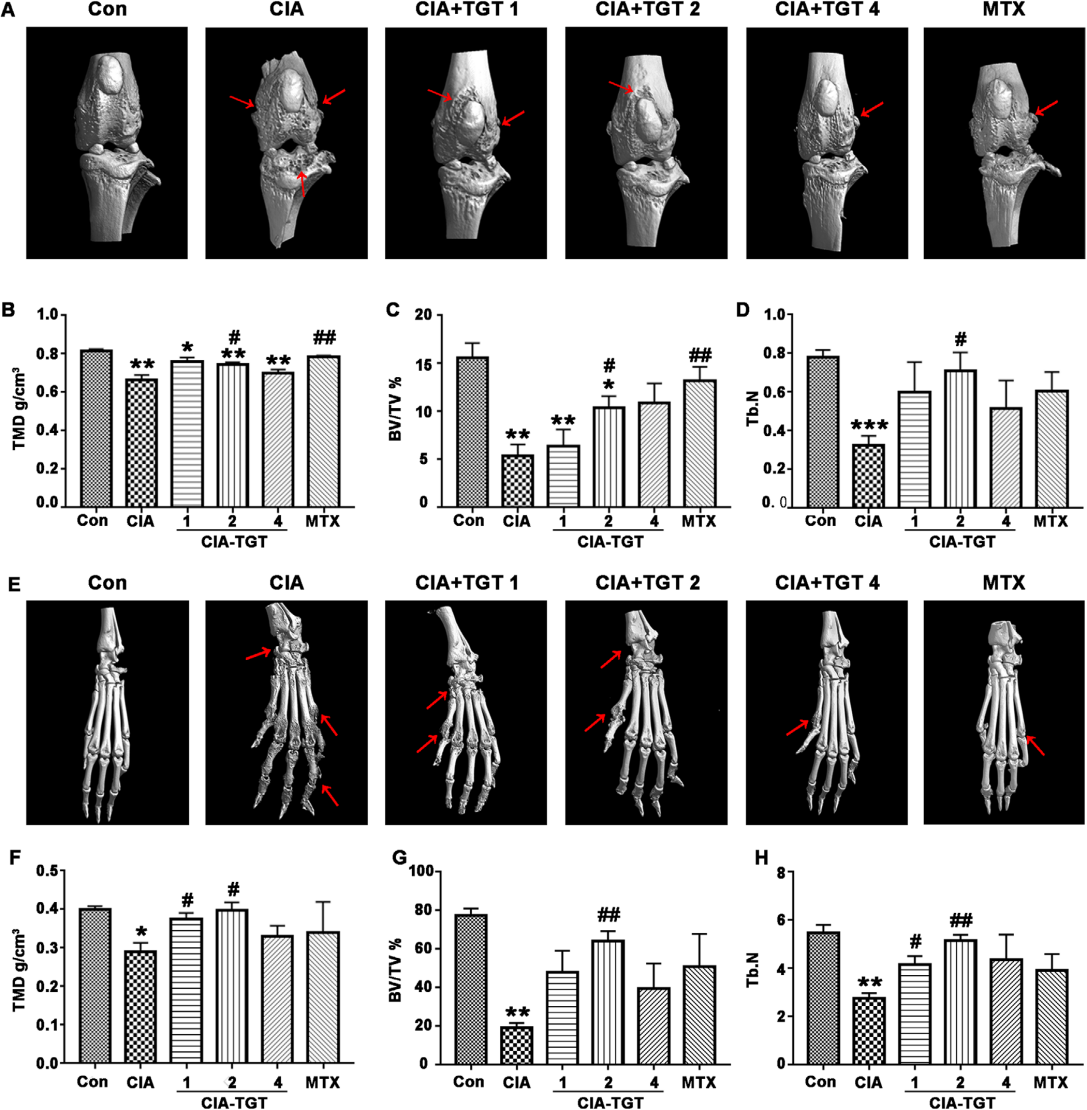
TGT treatment prevents bone destruction in CIA mice determined by micro‐CT scan. (A, E) Representative Micro‐CT images of knees and paws in different groups. The red arrows refer to bone erosion in CIA mice. (B‐D) Proximal tibia and (E‐H) calcaneus quantitative statistical analysis on the values of three parameters including tissue mineral density (TMD), bone volume fraction (BV/TV), and trabecular number (Tb.N), respectively. Data are expressed as mean ± SEM (n = 4); ^*^ *P *< .05, ^**^ *P *< .01, and ^***^ *P *< .001, compared with the normal control group; ^#^ *P *< .05 and ^##^ *P *< .01, compared with the CIA group

Micro‐CT scan was performed to evaluate the effects of TGT on protection of bone destruction in CIA mice. Compared with the normal mice, CIA mice showed enlarged joint space, massive joint destruction, and severe bone loss in both knees and paws (Figure 6A and E). Following the administration of TGT, the severity of bone erosions of CIA mice was markedly attenuated, especially CIA‐TGT‐2 (26 mg/kg) and CIA‐TGT‐4 (52 mg/kg) groups (Figure 6A and E), which was consistent with the quantitative evaluation on TMD, BV/TV and Tb.N in different groups of proximal tibia (Figure 6B‐D) and calcaneal (Figure 6F‐H).

#### 3.3.4 Differential expression patterns and the predictive efficiency of the ENST00000494760‐miR‐654‐5p‐C1QC axis in TGT response based on CIA mice

The above data confirmed the pharmacological actions of TGT against RA based on the CIA mouse model. Among the three dosages (13, 26, and 52 mg/kg), we found that the antiarthritis effects of 26 mg/kg TGT (CIA‐TGT‐2 group) were similar to that of 52 mg/kg TGT (CIA‐TGT‐4 group), which showed the obvious toxicity such as the decreased body weight of CIA mice in CIA‐TGT‐4 group (Figure 4D). Therefore, the CIA‐TGT‐2 group was selected for the following studies.

**FIGURE 7:**
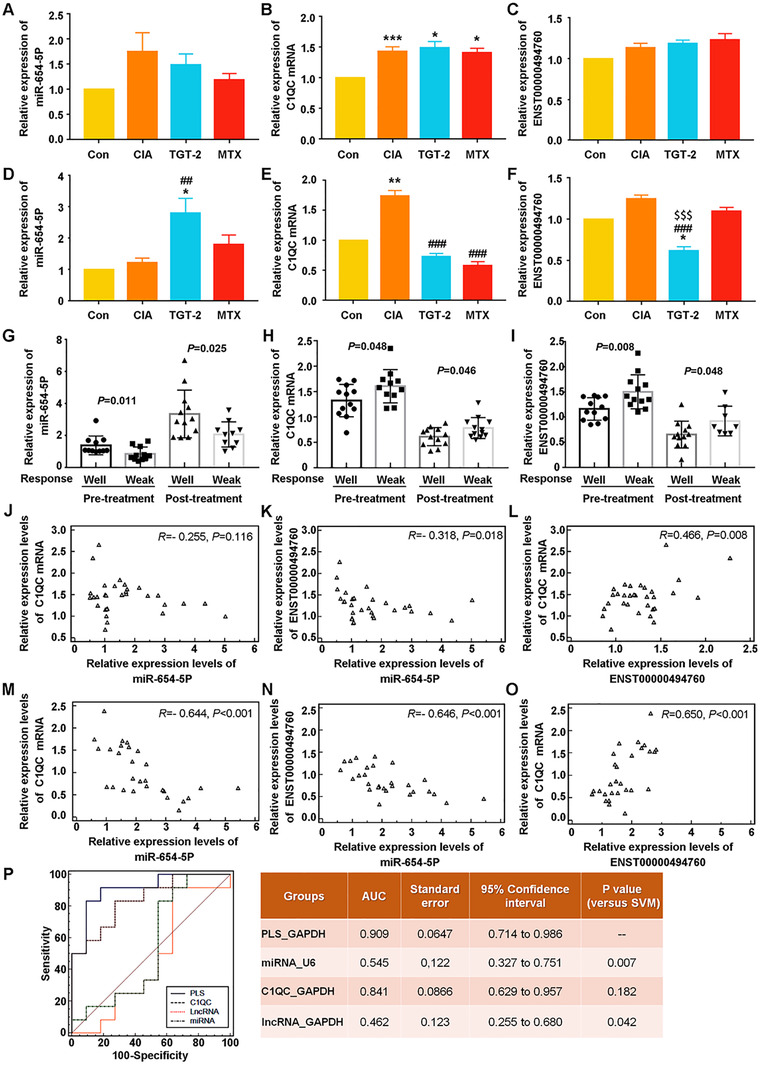
Differential expression patterns and the predictive efficiency of the ENST00000494760‐miR‐654‐5p‐C1QC axis in TGT response based on CIA mice. (A‐C) Expression levels of miR‐654‐5p, C1QC mRNA, and ENST00000494760 in normal and CIA mice in different groups before the treatment of TGT, respectively. (D‐F) Expression levels of miR‐654‐5p, C1QC mRNA, and ENST00000494760 in the peripheral blood samples of normal and CIA mice in different groups 2 weeks after the treatment of TGT, respectively. (G‐I) Comparison of miR‐654‐5p, C1QC mRNA, and ENST00000494760 expression between the TGT‐responder and ‐nonresponder groups, respectively. (J‐L) The correlation graphs of miR‐654‐5p‐C1QC mRNA, miR‐654‐5p‐ENST00000494760, and C1QC mRNA‐ENST00000494760 pairs before the treatment of TGT, respectively. (M‐O) The correlation graphs of miR‐654‐5p‐C1QC mRNA, miR‐654‐5p‐ENST00000494760, and C1QC mRNA‐ENST00000494760 pairs 2 weeks after the treatment of TGT, respectively. (P) ROC comparison of the PLS model which integrated the three RNAs and the single RNA biomarker in predicting TGT response. GAPDH was used as an internal control for the detection of CIQC mRNA and ENST00000494760 expression, while U6 was used as an internal control for the detection of miR‐654‐5p expression. Data are expressed as mean ± SEM (Con, n = 5; CIA‐TGT‐1, ‐2 and ‐4, n = 10 per group; Response‐well, n = 11; Response‐weak, n = 11);^*^ *P * < .05,^**^ *P*  < .01, and ^***^ *P * < .001, compared with the normal control group. ^##^ *P* < .01 and ^###^ *P*  < .001, compared with the CIA group. ^$$$^ *P*  < .001, compared with the MTX group

To verified the expression patterns and the associations of the ENST00000494760‐miR‐654‐5p‐C1QC axis with TGT response, we performed quantitative PCR analysis to detect the serum levels of the three RNAs in CIA mice before (Figure 7A‐C) and after (Figure 7D‐F) the treatment of TGT with the dosage of 26 mg/kg. After CIA modeling, C1QC mRNA expression in sera samples of CIA mice was dramatically higher than normal mice (*P *< .001, Figure 7B). However, the differences in miR‐654‐5p and lncRNA expression showed no statistical significant (Figure 7A and C). Following the treatment of TGT, the expression levels of CIQC mRNA and ENST00000494760 were both significantly reduced, while the miR‐654‐5p expression was dramatically increased (all *P *< .001, Figure 7D‐F).

The differences between the arthritis scores at 2 weeks after the TGT treatment and on the first day of TGT treatment were calculated. As a result, a total of 22 CIA mice in CIA‐TGT‐2 group were divided into TGT‐responder (well, n = 11, the differences of arthritis scores in the 2 time points were bigger than zero) and TGT‐nonresponder (weak, n = 11, the differences of arthritis scores in the 2 time points were smaller than zero) groups. The data before and after the treatment of TGT demonstrated that miR‐654‐5p expression in CIA‐TGT‐responders was significantly higher than that in CIA‐TGT‐nonresponders, while C1QC mRNA and ENST00000494760 expression in CIA‐TGT‐responders were markedly decreased compared with CIA‐TGT‐nonresponders (all *P *< .05, Figure 7G‐I).

In line with the clinical findings, both C1QC mRNA and ENST00000494760 were positively coexpressed in CIA‐TGT‐2 mice and negatively with the expression level of miR‐654‐5p before (Figure 7J‐L) and after (Figure 7M‐O) the treatment of TGT. Especially, the negative correlation between ENST00000494760 and miR‐654‐5p expression was more significant than that between C1QC mRNA and miR‐654‐5p expression before the TGT administration.

Moreover, the performance of our PLS model for predicting RA patient's response to TGT was further evaluated using the expression data of the three RNAs in peripheral blood of CIA mice in TGT‐responder and ‐nonresponder groups. The AUC value and the predictive accuracy of the PLS model were 0.909 and 83.33%, respectively. The ROC comparison showed that the AUC value of the PLS model which was integrated miR‐654‐5p, C1QC mRNA, and ENST00000494760 expression levels were dramatically higher than that of miR‐654‐5p or ENST00000494760 alone (both *P *< .05, Figure 7P). Although the AUC value of C1QC mRNA was lower than that of the PLS model, the differences had no statistical significance.

#### 3.3.5 Validation of the regulatory mechanism of the ENST00000494760‐miR‐654‐5p‐C1QC axis and its association with TGT response based on CIA mice in vivo

**FIGURE 8:**
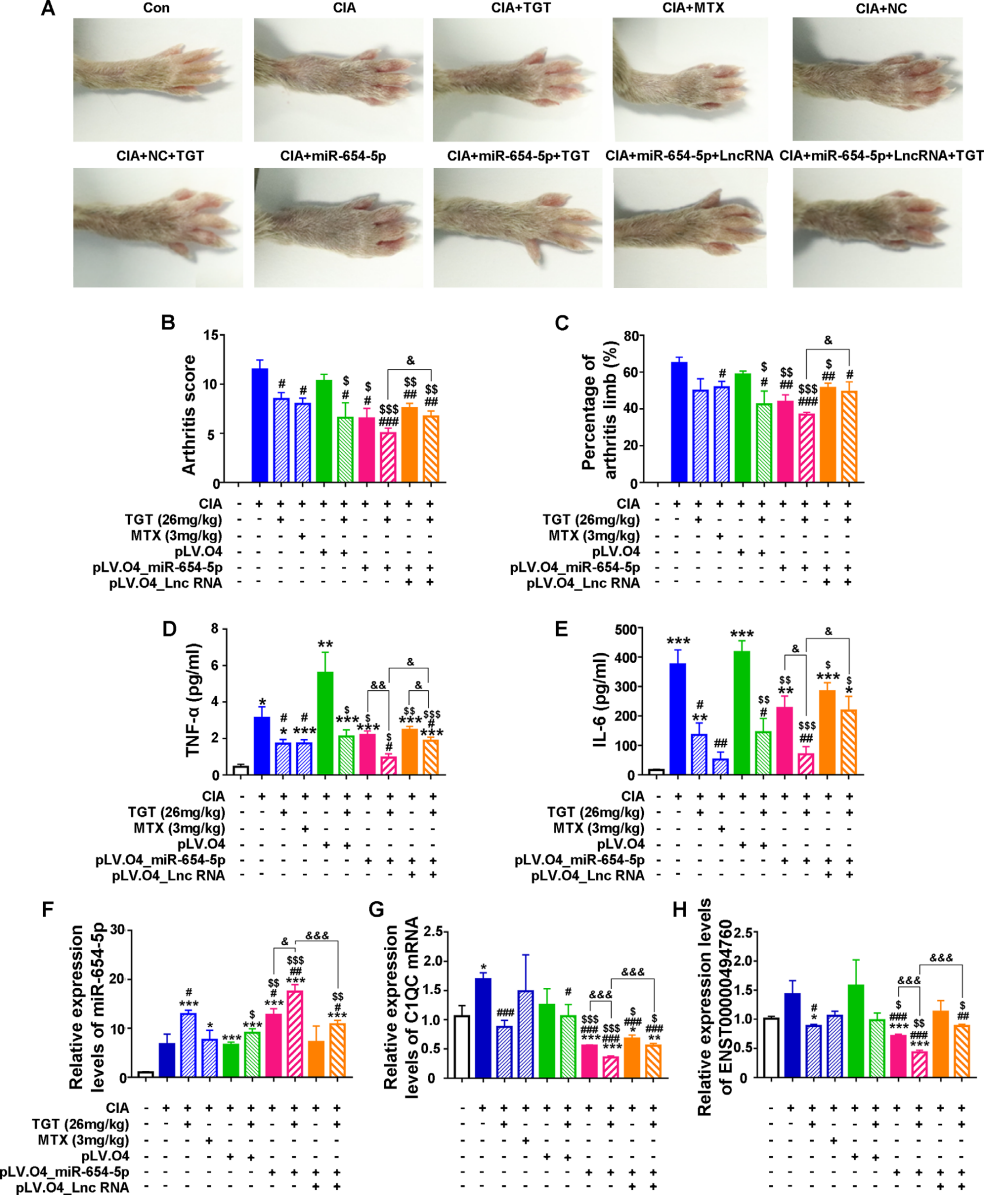
Validation of the regulatory mechanism of the ENST00000494760‐miR‐654‐5p‐C1QC axis and its association with TGT response based on CIA mice. (A) Macroscopic evidence of arthritis in different groups. (B) Arthritis score. (C) The percentage of the arthritis limbs. (D and E) Serum levels of TNF‐α and IL‐6 in different groups examined by ELISA. (F‐H) Expression levels of miR‐654‐5p, C1QC mRNA, and ENST00000494760 in the peripheral blood samples of normal and CIA mice in different groups detected by quantitative PCR analysis, respectively. GAPDH was used as an internal control for mRNA and lncRNA, while U6 was used as an internal control for miR‐654‐5p.*P  < .05, ***P  < .001, compared with the blank control. ^#^ *P*  < .05, ^##^ *P*  < .01, compared with CIA group. ^$^ *P *< .05, ^$$^ *P*  < .01, ^$$$^ *P*  < .001, compared with CIA‐miR‐NC group. ^&^ *P *< .05, ^&&^ *P* < .01,^&&&^ *P* < .001, comparison between the line marked groups

Two CIA mouse models with the overexpression of miR‐654‐5p, and the coexpression of ENST00000494760 and miR‐654‐5p were established using the lentivirus‐mediated delivery system, and then received the administration of TGT. The macroscopic evidence of arthritis (Figure 8A), the arthritis scores (Figure 8B), the percentage of arthritis limbs (Figure 8C), and the serum levels of proinflammatory cytokines (Figure 8D and E) all demonstrated that the pharmacological effects of TGT in miR‐654‐5p‐overexpressed CIA mice were better than that with cotransfection of ENST00000494760 and miR‐654‐5p expression. Mechanically, the enforced miR‐654‐5p expression effectively suppressed the expression of C1QC mRNA and ENST00000494760 in CIA mice which improved the pharmacological effects of TGT, while the cotransfection of ENST00000494760 and miR‐654‐5p expression vectors dramatically reduced the inhibitory effect of miR‐654‐5p on C1QC mRNA expression leading to the weak response to TGT administration (Figure 8F‐H).

#### 3.3.6 Validation of the regulatory mechanism of the ENST00000494760‐miR‐654‐5p‐C1QC axis and its association with TGT response based on MH7A cells in vitro

**FIGURE 9:**
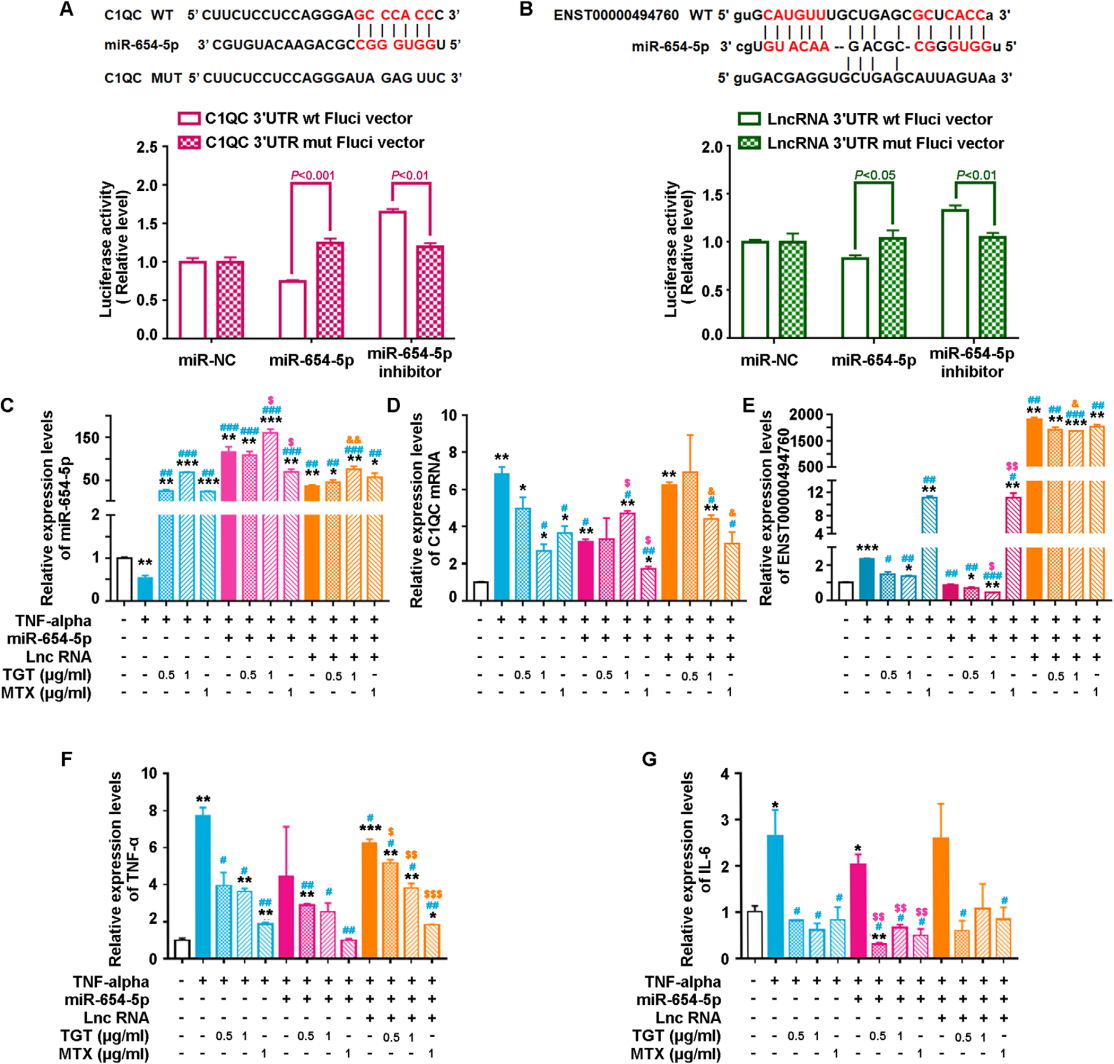
Validation of the regulatory mechanism of the ENST00000494760‐miR‐654‐5p‐C1QC axis and its association with TGT response based on MH7A cells.(A) The binding sites within the seed region sequence in the 3′‐UTR of C1QC mRNA to miR‐654‐5p and the luciferase activities of MH7A cells in different groups. (B) The binding sites within the seed region sequence in the 3′‐UTR of ENST00000494760 to miR‐654‐5p and the luciferase activities of MH7A cells in different groups. (C‐E) Expression levels of miR‐654‐5p, C1QC mRNA, and ENST00000494760 in MH7A cells of different groups detected by quantitative PCR analysis, respectively. β‐actin was used as an internal control for mRNA and lncRNA, while U6 was used as an internal control for miR‐654‐5p. *P  < .05, ***P  < .001, compared with the blank control. ^#^ *P*  < .05, ^##^ *P * < .01, ^###^ *P*  < .001, compared with TNF‐α induced group. ^$^ *P *< .05, ^$$^ *P*  < .001, compared with miR‐654‐5p mimics‐transfection group. ^&^ *P *< .05, compared with miR‐654‐5p mimics and ENST00000494760 expression vector cotransfection group. (F, G) Expression levels of TNF‐α and IL‐6 mRNAs in MH7A cells of different groups detected by quantitative PCR analysis, respectively. β‐actin was used as an internal control for mRNA and lncRNA, while U6 was used as an internal control for miR‐654‐5p. *P  < .05, ***P  < .001, compared with the blank control. ^#^ *P*  < .05, ^##^ *P*  < .01, compared with TNF‐α induced group. ^$^ *P *< .05, ^$$^ *P * < .001, compared with miR‐654‐5p mimics‐transfection group

C1QC mRNA and ENST00000494760 were predicted as the common targets of miR‐654‐5p by both TargetScan and miRanda. miR‐654‐5p harbors the complementary binding sequences of both C1QC mRNA and ENST00000494760 (Figure 9A and B). To validate this hypothesis, we performed a luciferase reporter assay to investigate the correlations between C1QC mRNA and miR‐654‐5p, and between ENST00000494760 and miR‐654‐5p. Cotransfection of C1QC‐3′UTR‐WT and miR‐654‐5p mimics into the human RA synovial MH7A cells resulted into a mark reduction of the luciferase activity compared with that from the cotransfection of C1QC‐3′UTR‐MUT and miR‐654‐5p (*P *< .001, Figure 9A). In contrast, the luciferase activity of MH7A cells with the cotransfection of C1QC‐3′UTR‐WT and miR‐654‐5p inhibitor was markedly higher than that with the cotransfection of C1QC‐3′UTR‐MUT and miR‐654‐5p inhibitor (*P *< .01, Figure 9A). The similar results were found in the luciferase reporter assay on the correlations between ENST00000494760 and miR‐654‐5p (both *P *< .05, Figure 9B).

Additionally, the enforced expression of miR‐654‐5p significantly suppressed C1QC mRNA and ENST00000494760 expression levels in MH7A cells induced by TNF‐α, while the cotransfection of ENST00000494760 expression vector and miR‐654‐5p mimics dramatically reduced the inhibitory effect of miR‐654‐5p on C1QC mRNA expression (both *P *< .05, Figure 9C‐E). Moreover, the administration of TGT and the enforced expression of miR‐654‐5p synergistically reduced C1QC mRNA and ENST00000494760 expression in MH7A cells induced by TNF‐α, but the introduction of ENST00000494760 suppressed this synergy (all *P *< .05, Figure 9C‐E).

Moreover, the expression levels of IL‐6 and TNF‐α mRNAs stimulated by TNF‐α in MH7A cells with or without the RNA transfection and the treatment of TGT were detected by quantitative PCR analysis. As shown in Figure 9F‐G, the mRNA levels of the proinflammatory cytokine expression were markedly elevated by the stimulation of TNF‐α, which were abolished by TGT at a concentration ranging from 0.5 to 1.0 µg/mL (all *P *< .05). In line with the effects of TGT on the RNA expression, the administration of TGT and the enforced expression of miR‐654‐5p synergistically decreased the expression levels of IL‐6 and TNF‐α mRNAs stimulated by TNF‐α in MH7A cells, but the introduction of ENST00000494760 suppressed this synergy (Figure 9F and G).

## 4 DISCUSSION

Accumulating clinical observations show that there are approximately 30% nonresponders of RA patients to TGT treatment, which may be a constraining factor in effective therapy of RA and clinical application of TGT. However, the mechanisms underlying individual differences in response to TGT remain poorly understood. Crosstalks between ceRNAs mediated by common miRNAs have been revealed to play crucial roles both in normal physiology and pathological processes.^14^ Therefore, the novel identified ceRNA axis from RNA coexpression modules and regulatory networks associated with clinical traits may provide new insight for clinical management, and also be attractive for systems‐level decoding of precision medicine. In the current study, our bioinformatics analysis predicted the ENST00000494760‐miR‐654‐5p‐C1QC axis to be a candidate biomarker for RA patients' response to TGT according to RNA expression profilings, lncRNA‐mRNA coexpression network, and ceRNA network. After that, we verified the differential expression characteristics of ENST00000494760, miR‐654‐5p, and C1QC mRNA in peripheral blood of TGT responders and nonresponders based on a large independent validation cohort and CIA mice. To determine the significance of the ENST00000494760‐miR‐654‐5p‐C1QC axis in RA patients' response to TGT, the PLS‐based prediction model of TGT responder was constructed using the expression levels of three RNAs and the independent dataset test demonstrated the favorable predictive performance of this model, in accord with our findings based on the CIA mouse model. Further luciferase reporter gene assay verified that miR‐654‐5p directly bound to the 3′‐untranslated region of both ENST00000494760 and C1QC mRNA to inhibit their expression in human RA synovial MH7A cells. Mechanistically, miR‐654‐5p suppressed the expression of C1QC mRNA, but ENST00000494760 bound to miR‐654‐5p and relieved its repression on C1QC mRNA, leading to the aggressive progression of RA and the weak response to TGT treatment. These findings shed light on the significance of miR‐654‐5p‐mediated ceRNA interactions in screening RA patients who may get benefits from TGT treatment, and also provide an important clue for ceRNA network‐based approach to accelerate development of personalized medicine.

miR‐654‐5p, mapped to chromosome 14q32.31, was originally identified from the miRNA microarray based on prostate cancer tissues and was verified to function as a stronger inhibitor of tumor growth.^15^ Recently, it has been observed to be deregulated and play crucial roles in a variety of cancers.^16^, ^17^, ^18^, ^19^, ^20^ Despite accumulating research on its involvement in carcinogenesis and cancer management, this is the first study to identify the associations of miR‐654‐5p with RA response to TGT treatment. In the current study, our microarray data based on the discover cohort and independent test data based on the large validation cohort both verified the significant upregulation of miR‐654‐5p in peripheral blood of TGT‐responders compared to nonresponders, in coincided with the findings based on CIA mice. The comparison of the qPCR data before and after TGT treatment based on both CIA mice and MH7A cells showed that TGT effectively enhanced the expression of miR‐654‐5p.

To date, the downstream miR‐654‐5p dependent signaling has not been fully elucidated. EPSTI1 for breast cancer,^16^ AR for prostate cancer,^15^ GRAP for oral squamous cell carcinoma,^17^ CDCP1 and PLAGL2 for ovarian cancer were the only five direct targets identified for miR‐654‐5p thus far. In the present study, we demonstrated that both C1QC mRNA and ENST00000494760 were direct targets of miR‐654‐5p, and shared the same miRNA response elements, leading to the ceRNA function of ENST00000494760, which exerts its decoy activity by recruiting miR‐654‐5p via basepairing with miRNA‐recognition elements, subsequently causing release of C1QC from miRNA control. C1QC gene encodes the C‐chain polypeptide of serum complement subcomponent C1q and is involved into the classical pathway of complement activation (from the human gene database‐Gene Cards). C1QC is one of polypeptide chains of C1q. C1q deficiency has been indicated to be related with the increased risk for developing autoimmunity. Notably, C1QC is one of the targets hit by Etanercept and Adalimumab, which are both FDA‐approved anti‐RA agents (DrugBank database). Teo et al^21^ firstly reported the C1q production by osteoclasts and its ability to enhance osteoclast development. Similarly, our data demonstrated that C1QC mRNA expression were effectively reduced by TGT. Additionally, RA patients with low C1QC levels may display good response to TGT treatment. We also evaluated the antiarthritic effect of TGT using CIA model of mice. Interestingly, TGT effectively ameliorated the clinical and serological features of CIA mice, and especially reduced the histological scores and destruction of cartilage and bone. Moreover, our experimental data demonstrated the decreased expression of C1QC mRNA in CIA mice and TNF‐alpha‐simulated MH7A cells following the treatment of TGT, subsequently result in reducing the production of multiple inflammatory cytokines. More importantly, the enforced expression of miR‐654‐5p significantly reduced C1QC mRNA expression, which was synergistically to the effects of TGT. In contrast, the cotransfection of ENST00000494760 and miR‐654‐5p expression vectors in both CIA mice and TNF‐alpha‐simulated MH7A cells displayed a decreasing impact on anti‐inflammatory effects of TGT. These results confirmed our clinical findings, that is, miR‐654‐5p upregulation, combined with C1QC and ENST00000494760 downregulation may indicate the good response of TGT treatment.

In conclusion, our findings suggest that the overexpressed lncRNA ENST00000494760 in RA may sponge miR‐654‐5p to promote C1QC expression. This novel ceRNAaxis may modulate the balance of inflammatory‐immune system of RA patients and may serve as a biomarker for screening the responsive RA patients to TGT treatment, which will allow improved personalized healthcare.

## CONFLICT OF INTEREST

The authors do not have any conflict of interest with the content of the manuscript.

## AUTHORS' CONTRIBUTION

ZY designed the study, performed the data analysis, and drafted the manuscript. LN conceived of the study, participated in its design, and reviewed the manuscript. WX carried out the experimental validation based on CIA mice and drafted the part of manuscript. The other authors participated in the clinical sample collection and performed the statistical analysis. All authors read and approved the final manuscript.

5

## Supporting information

Supporting information

Supporting information

## Data Availability

The data that supports the findings of this study are available in the supplementary material of this article.
