# Supplementary material for: Inferences of individual differences in response to tripterysium glycosides across patients with Rheumatoid arthritis using a novel ceRNA regulatory axis
Source: Clin Transl Med. 2020 Oct 9;10(6):e185. doi: 10.1002/ctm2.185 (PMC7545341; doi:10.1002/ctm2.185)
Supplement: Supplementary file 1 — Supporting information [file CTM2-10-e185-s001.pdf]

Supplementary file for

" Inferences of Individual Differences in Response to Tripterysium Glycosides across Patients with Rheumatoid Arthritis Using a Novel ceRNA Regulatory Axis "

Supplementary Figure

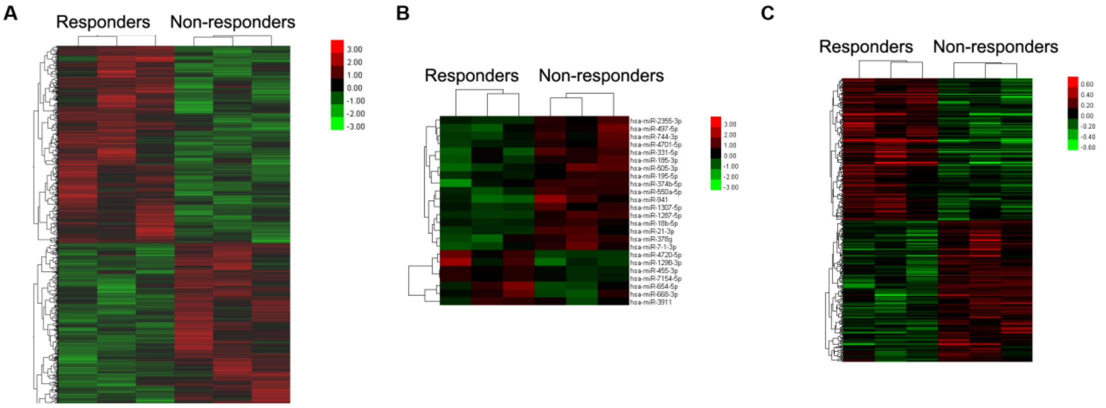

Figure S1 Differentially expressed lncRNAs (A), miRNAs (B) and mRNAs (C) associated with the patients' response to TGT. Heat map showing hierarchical clustering of mRNAs and miRNAs, whose expression changes were more than 1.5-fold in the comparison between the responder and non-responder groups. In clustering analysis, up- and down-regulated genes are colored in red and green, respectively.

Table S1 Detailed information of clinical and inflammatory parameters of RA patients enrolled in the current study

Case No.	Groups	Age (Years)	Gender	RF (U/mL)	Anti-CCP (IU/mL)	ESR (mm)	CRP (mg/dL)
			**Discovery cohort**				
**1**	Non-responder	45	Male	＜20	＜25	81	83.72
**2**	Non-responder	51	Female	115	50.39	70	27.81
**3**	Responder	52	Female	39.9	--	19	1.85
**4**	Responder	51	Male	--	--	--	--
**5**	Non-responder	55	Female	＜20	2829	57	32.36
**6**	Responder	52	Female	63.8	926.67	71	4.46
			**Validation cohort**				
**1**	Responder	58	Male	417	823.74	9	3.65
**2**	Non-responder	58	Male	68.1	＜25	60	76.65
**3**	Non-responder	51	Male	278	3111.03	13	10.06
**4**	Non-responder	62	Female	＜20	＜25	22	3
**5**	Non-responder	66	Female	--	58.42	--	--
**6**	Non-responder	61	Female	163	1435.32	57	20.90
**7**	Responder	65	Female	45.6	--	13	4.52

**8**	Non-responder	60	Female	105	2321.58	25	5
**9**	Responder	65	Male	262	--	38	18.56
**10**	Non-responder	55	Female	1120	--	86	12.51
**11**	Non-responder	53	Female	117	--	14	4.3
**12**	Responder	76	Female	325	--	44	6.39
**13**	Responder	65	Female	20.9	1978.21	17	1
**14**	Responder	49	Female	70.3	--	28	12.47
**15**	Responder	69	Female	29.4	470.72	31	1.43
**16**	Responder	74	Female	--	--	79	44.65
**17**	Non-responder	50	Female	831	37.82	--	20
**18**	Non-responder	56	Female	--	--	87	18
**19**	Responder	49	Female	--	--	--	1
**20**	Responder	66	Female	447	2148.21	23	3.35
**21**	Non-responder	84	Male	＜20	--	92	177.62
**22**	Responder	66	Female	25.6	2176.7	81	22.53
**23**	Responder	62	Male	＜20	--	29	5.86
**24**	Non-responder	60	Female	＜20	--	73	2.83

**25**	Non-responder	48	Female	--	40.07	71	--
**26**	Responder	62	Female	1010	--	17	6.13
**27**	Non-responder	33	Male	--	--	10	17
**28**	Responder	55	Female	270	--	28	20.37
**29**	Responder	25	Male	--	--	23	91.10
**30**	Non-responder	51	Female	＜20	＜25	44	12.07
**31**	Non-responder	40	Female	503	--	19	1
**32**	Responder	56	Female	＜20	＜25	19	-
**33**	Responder	78	Female	888	-	106	59.04
**34**	Responder	70	Female	530	851.23	13	-
**35**	Responder	60	Male	-	＜25	-	5.92
**36**	Responder	58	Female	＜20	48.71	16	1.00
**37**	Responder	68	Female	177	3015.65	8	-
**38**	Responder	54	Female	＜20	＜25	8	-
**39**	Responder	74	Male	＜20	-	25	-
**40**	Non-responder	55	Female	＜20	＜25	34	8.54
**41**	Non-responder	53	Female	682	664.44	90	-

**42**	Responder	60	Male	165	-	13	-
**43**	Responder	62	Male	-	-	-	5.91
**44**	Responder	63	Female	970	＜25	45	1.09
**45**	Responder	48	Female	＜20	183.52	33	11

Table S2 All sequences of the primers listed in the 5’-3’ direction

Species	RNA Name	Sequence: 5’-3’
Homo species (Peripheral Blood)	18S	F: GCGGCGGAAAATAGCCTTTG
		R: GATCACACGTTCCACCTCATC
	GAPDH	F: GGAGCGAGATCCCTCCAAAAT
		R: [1]GGCTGTTGTCATACTTCTCATGG
	C1QC	F: AGGATGGGTACGACGGACTG
		R: GTAAGCCGGGTTCTCCCTTC
	*U6	Art.No.: CD201-0145 (TIANGEN BIOTECH)
	*miR-654-5p	F: TGGTGGGCCGCAGAACAT
	ENST00000494760	F: CCCTCTTTCCTTGGTCGTGG
		R: TCACAGGAACTGGGCATGGA
Homo species (MH7A cell)	IL-6	F：TGGATTCAATGAGGAGACTTGC<br>R: TCAGGGGTGGTTATTGCATCT
	TNF-alpha	F：GGACCTCTCTCTAATCAGCCC<br>R: TCAGCTTGAGGGTTTGCTAC
	IL-1beta	F：TGAGCTCGCCAGTGAAATGA<br>R: AGGAGCACTTCATCTGTTTAGGG

	IL-8	F：ACACTGCGCCAACACAGAAA<br><br>R: AACTTCTCCACAACCCTCTGC
	β-actin	F：AGCGAGCATCCCCCAAAGTT
		R：GGGCACGAAGGCTCATCATT
	C1QC	F：AAAGTCCCCGGCCTCTACTA
		R:TCATTGACAGCCAGCCACAC
	ENST00000494760	F：TCCACTAGAAGCGAGGTGTGT
		R: TTTTGTGAGTGCCTCCTACCT
	miRNA-654-5p*	F：TGGGCCGCAGAACATGTG
	U6*	F：GCAAATTCGTGAAGCGTTCC
Mus musculus (Blood)	GAPDH	F: AGGTCGGTGTGAACGGATTTG
		R: GGGGTCGTTGATGGCAACA
	C1QC	F: GATCCATCGGCCCTGTATCTC
		R: TATTTCTCTATCTCACCACCTGAAC
	U6	F: GCTTCGGCAGCACATATACTAAAAT
		R: CGCTTCACGAATTTGCGTGTCAT
	*miR-654-5p	F: GGCTGTATTCCCCTCCATCG
	ENST00000494760	F: TCCACTAGAAGCGAGGTGTGT
		R: TTTTGTGAGTGCCTCCTACCT

*According to manual, we choose entire sequence of miR-654-5p or U6 as miRNA specific 5’ primer. The 3’ primer for qPCR is the mRQ 3’Primer with kit (638313, TaKara, Kusatsu, Japan)

Table S3 The relative degree differences of RNAs in lncRNA-mRNA coexpression networks between TGT-responder and TGT-non-responder groups

Gene symbol/LcRNA	Responder_Degree	Responder_Relative Degree	Non-responder_Degree	Non-responder_Relative Degree	|Relative degree differences|
CYP2B6	38	0.95	22	0.511627907	0.438372093
NONHSAT041902	37	0.925	21	0.488372093	0.436627907
ENST00000420617	37	0.925	22	0.511627907	0.413372093
NONHSAT123539	37	0.925	22	0.511627907	0.413372093
NAB1	36	0.9	21	0.488372093	0.411627907
ENST00000436582	36	0.9	21	0.488372093	0.411627907
ENST00000494760	34	0.85	19	0.441860465	0.408139535
ENST00000460672	38	0.95	24	0.558139535	0.391860465
TXNDC12	36	0.9	22	0.511627907	0.388372093
NONHSAT083226	22	0.55	40	0.930232558	0.380232558
C1QC	38	0.95	25	0.581395349	0.368604651
ENST00000585828	37	0.925	24	0.558139535	0.366860465
TIFAB	35	0.875	22	0.511627907	0.363372093
AMOT	34	0.85	21	0.488372093	0.361627907

SLC35E3	34	0.85	21	0.488372093	0.361627907
ENST00000580914	32	0.8	19	0.441860465	0.358139535
ASTL	40	1	28	0.651162791	0.348837209
GNL3L	38	0.95	26	0.604651163	0.345348837
GPR82	31	0.775	19	0.441860465	0.333139535
APOA5	31	0.775	19	0.441860465	0.333139535
ENST00000485975	40	1	29	0.674418605	0.325581395
CCDC57	27	0.675	43	1	0.325
TRHR	38	0.95	27	0.627906977	0.322093023
CDK10	38	0.95	27	0.627906977	0.322093023
FOXF2	36	0.9	25	0.581395349	0.318604651
CCDC126	36	0.9	25	0.581395349	0.318604651
GJB7	32	0.8	21	0.488372093	0.311627907
SLC22A20	32	0.8	21	0.488372093	0.311627907
ENST00000593706	36	0.9	26	0.604651163	0.295348837
ENST00000471085	34	0.85	24	0.558139535	0.291860465
ENST00000417539	31	0.775	21	0.488372093	0.286627907

ZNF273	31	0.775	21	0.488372093	0.286627907
ENST00000474370	31	0.775	21	0.488372093	0.286627907
ENST00000474664	31	0.775	21	0.488372093	0.286627907
CDH8	27	0.675	41	0.953488372	0.278488372
ENST00000556386	39	0.975	30	0.697674419	0.277325581
UGT2B4	39	0.975	30	0.697674419	0.277325581
NONHSAT008282	38	0.95	29	0.674418605	0.275581395
ADH7	36	0.9	27	0.627906977	0.272093023
ZNF536	34	0.85	25	0.581395349	0.268604651
UCKL1	34	0.85	25	0.581395349	0.268604651
ENST00000580063	33	0.825	24	0.558139535	0.266860465
HOXB6	32	0.8	23	0.534883721	0.265116279
ENST00000471094	32	0.8	23	0.534883721	0.265116279
ENST00000583051	22	0.55	35	0.813953488	0.263953488
ACMSD	22	0.55	35	0.813953488	0.263953488
ISY1	31	0.775	22	0.511627907	0.263372093
MAGEA12	31	0.775	22	0.511627907	0.263372093

TEX28	30	0.75	21	0.488372093	0.261627907
ENST00000474793	30	0.75	21	0.488372093	0.261627907
DMRT2	24	0.6	37	0.860465116	0.260465116
NPAS2	24	0.6	37	0.860465116	0.260465116
KRT20	29	0.725	20	0.465116279	0.259883721
ENST00000587524	27	0.675	40	0.930232558	0.255232558
NHP2L1	38	0.95	30	0.697674419	0.252325581
ENST00000492808	36	0.9	28	0.651162791	0.248837209
ENST00000441316	36	0.9	28	0.651162791	0.248837209
ENST00000366099	36	0.9	28	0.651162791	0.248837209
ARHGAP28	36	0.9	28	0.651162791	0.248837209
GPRC5B	36	0.9	28	0.651162791	0.248837209
WDR72	35	0.875	27	0.627906977	0.247093023
GABRA6	35	0.875	27	0.627906977	0.247093023
ENST00000490564	34	0.85	26	0.604651163	0.245348837
ENST00000524554	34	0.85	26	0.604651163	0.245348837
ENST00000563133	34	0.85	26	0.604651163	0.245348837

HAND1	34	0.85	26	0.604651163	0.245348837
ENST00000410569	33	0.825	25	0.581395349	0.243604651
KLHDC8B	32	0.8	24	0.558139535	0.241860465
NONHSAT000636	30	0.75	22	0.511627907	0.238372093
DMRT1	24	0.6	36	0.837209302	0.237209302
GLB1L3	25	0.625	37	0.860465116	0.235465116
ATP1A3	25	0.625	37	0.860465116	0.235465116
ENST00000484985	25	0.625	37	0.860465116	0.235465116
GAL3ST3	25	0.625	37	0.860465116	0.235465116
ZC3H10	38	0.95	31	0.720930233	0.229069767
CD80	38	0.95	31	0.720930233	0.229069767
ENST00000461340	37	0.925	30	0.697674419	0.227325581
TIMM8A	30	0.75	42	0.976744186	0.226744186
RSL24D1	36	0.9	29	0.674418605	0.225581395
ENST00000549080	35	0.875	28	0.651162791	0.223837209
DDTL	34	0.85	27	0.627906977	0.222093023
ENST00000536112	34	0.85	27	0.627906977	0.222093023

ENST00000476458	32	0.8	25	0.581395349	0.218604651
SERPINH1	23	0.575	34	0.790697674	0.215697674
ENST00000566103	23	0.575	34	0.790697674	0.215697674
ENST00000505982	30	0.75	23	0.534883721	0.215116279
STARD3NL	24	0.6	35	0.813953488	0.213953488
ENST00000567228	24	0.6	35	0.813953488	0.213953488
CDX1	26	0.65	37	0.860465116	0.210465116
RNLS	26	0.65	37	0.860465116	0.210465116
SARM1	40	1	34	0.790697674	0.209302326
NONHSAT129059	27	0.675	38	0.88372093	0.20872093
ENST00000534281	37	0.925	31	0.720930233	0.204069767
NONHSAT082390	35	0.875	29	0.674418605	0.200581395
CASC3	35	0.875	29	0.674418605	0.200581395
RAB40A	34	0.85	28	0.651162791	0.198837209
PLN	33	0.825	27	0.627906977	0.197093023
ENST00000557660	33	0.825	27	0.627906977	0.197093023
BYSL	31	0.775	25	0.581395349	0.193604651

ENST00000517028	31	0.775	25	0.581395349	0.193604651
ELAVL1	31	0.775	25	0.581395349	0.193604651
ZNF660	23	0.575	33	0.76744186	0.19244186
ENST00000595383	25	0.625	35	0.813953488	0.188953488
TUB	25	0.625	35	0.813953488	0.188953488
ENST00000480447	26	0.65	36	0.837209302	0.187209302
NONHSAT007259	27	0.675	37	0.860465116	0.185465116
VPS37B	27	0.675	37	0.860465116	0.185465116
LEPREL1	27	0.675	37	0.860465116	0.185465116
FR0245974	28	0.7	38	0.88372093	0.18372093
OSGIN2	28	0.7	38	0.88372093	0.18372093
PPP4R4	38	0.95	33	0.76744186	0.18255814
ENST00000598814	38	0.95	33	0.76744186	0.18255814
RPAP1	38	0.95	33	0.76744186	0.18255814
NONHSAT122778	37	0.925	32	0.744186047	0.180813953
NRAS	35	0.875	30	0.697674419	0.177325581
DAGLA	32	0.8	42	0.976744186	0.176744186

ZNF410	34	0.85	29	0.674418605	0.175581395
FKBP9	33	0.825	28	0.651162791	0.173837209
NEK5	32	0.8	27	0.627906977	0.172093023
ASZ1	32	0.8	27	0.627906977	0.172093023
FAF1	32	0.8	27	0.627906977	0.172093023
YWHAE	22	0.55	31	0.720930233	0.170930233
GRAMD1C	31	0.775	26	0.604651163	0.170348837
ZBTB3	31	0.775	26	0.604651163	0.170348837
NONHSAT018233	30	0.75	25	0.581395349	0.168604651
ADC	24	0.6	33	0.76744186	0.16744186
CHRM2	29	0.725	24	0.558139535	0.166860465
ENST00000552294	28	0.7	23	0.534883721	0.165116279
MED10	27	0.675	36	0.837209302	0.162209302
ENST00000495131	38	0.95	34	0.790697674	0.159302326
ENST00000448869	38	0.95	34	0.790697674	0.159302326
ENST00000504426	37	0.925	33	0.76744186	0.15755814
ENST00000572721	30	0.75	39	0.906976744	0.156976744

ZNF573	36	0.9	32	0.744186047	0.155813953
ENST00000487378	31	0.775	40	0.930232558	0.155232558
ENST00000569390	31	0.775	40	0.930232558	0.155232558
ENST00000470741	35	0.875	31	0.720930233	0.154069767
NUDT22	32	0.8	41	0.953488372	0.153488372
BAI3	34	0.85	30	0.697674419	0.152325581
ENST00000524688	34	0.85	30	0.697674419	0.152325581
ENST00000487920	34	0.85	30	0.697674419	0.152325581
n409333	34	0.85	30	0.697674419	0.152325581
PDE9A	33	0.825	29	0.674418605	0.150581395
CDH6	33	0.825	29	0.674418605	0.150581395
LPAR3	32	0.8	28	0.651162791	0.148837209
ENST00000521361	32	0.8	28	0.651162791	0.148837209
ENST00000600795	31	0.775	27	0.627906977	0.147093023
FAM123B	29	0.725	25	0.581395349	0.143604651
ENST00000473426	26	0.65	34	0.790697674	0.140697674
NONHSAT057067	26	0.65	34	0.790697674	0.140697674

NPM1	27	0.675	35	0.813953488	0.138953488
HSPA2	39	0.975	36	0.837209302	0.137790698
FXYD3	28	0.7	36	0.837209302	0.137209302
ENST00000489988	28	0.7	36	0.837209302	0.137209302
PCYT1B	38	0.95	35	0.813953488	0.136046512
ENST00000524768	29	0.725	37	0.860465116	0.135465116
ENST00000334448	29	0.725	37	0.860465116	0.135465116
HERC2	37	0.925	34	0.790697674	0.134302326
TCTN1	30	0.75	38	0.88372093	0.13372093
ENST00000580267	30	0.75	38	0.88372093	0.13372093
ENST00000468914	35	0.875	32	0.744186047	0.130813953
NONHSAT064246	35	0.875	32	0.744186047	0.130813953
MRO	35	0.875	32	0.744186047	0.130813953
PHLDB2	32	0.8	40	0.930232558	0.130232558
ENST00000479300	33	0.825	30	0.697674419	0.127325581
GRM7	33	0.825	30	0.697674419	0.127325581
CREM	33	0.825	30	0.697674419	0.127325581

SELV	33	0.825	30	0.697674419	0.127325581
CYP4F2	33	0.825	30	0.697674419	0.127325581
CDH7	35	0.875	43	1	0.125
ENST00000504382	30	0.75	27	0.627906977	0.122093023
ENST00000525984	24	0.6	31	0.720930233	0.120930233
ENTHD1	29	0.725	26	0.604651163	0.120348837
NONHSAT091725	28	0.7	25	0.581395349	0.118604651
CDC42SE1	28	0.7	25	0.581395349	0.118604651
GPR115	28	0.7	25	0.581395349	0.118604651
NRN1	26	0.65	33	0.76744186	0.11744186
ENST00000596593	27	0.675	34	0.790697674	0.115697674
SNX33	27	0.675	34	0.790697674	0.115697674
CDC20B	26	0.65	23	0.534883721	0.115116279
SYT4	39	0.975	37	0.860465116	0.114534884
KIAA0284	28	0.7	35	0.813953488	0.113953488
SLC8A3	28	0.7	35	0.813953488	0.113953488
NONHSAT079699	28	0.7	35	0.813953488	0.113953488

NONHSAT076186	28	0.7	35	0.813953488	0.113953488
ENST00000471144	30	0.75	37	0.860465116	0.110465116
CAMK1D	30	0.75	37	0.860465116	0.110465116
ENST00000580086	30	0.75	37	0.860465116	0.110465116
PHKG1	36	0.9	34	0.790697674	0.109302326
ENST00000575253	35	0.875	33	0.76744186	0.10755814
NONHSAT082479	35	0.875	33	0.76744186	0.10755814
ENST00000467610	34	0.85	32	0.744186047	0.105813953
PTTG1IP	34	0.85	32	0.744186047	0.105813953
NONHSAT024904	33	0.825	40	0.930232558	0.105232558
XDH	33	0.825	40	0.930232558	0.105232558
CYP46A1	32	0.8	30	0.697674419	0.102325581
RTDR1	32	0.8	30	0.697674419	0.102325581
ENST00000462712	32	0.8	30	0.697674419	0.102325581
ZNF530	35	0.875	42	0.976744186	0.101744186
SIM1	35	0.875	42	0.976744186	0.101744186
NUDT10	22	0.55	28	0.651162791	0.101162791

ZNF35	31	0.775	29	0.674418605	0.100581395
NONHSAT001685	31	0.775	29	0.674418605	0.100581395
ENST00000588015	23	0.575	29	0.674418605	0.099418605
FR0374378	23	0.575	29	0.674418605	0.099418605
ENST00000579529	23	0.575	29	0.674418605	0.099418605
TCTN2	30	0.75	28	0.651162791	0.098837209
ENST00000435216	25	0.625	31	0.720930233	0.095930233
CCL19	28	0.7	34	0.790697674	0.090697674
GDPD2	28	0.7	34	0.790697674	0.090697674
BNIP1	25	0.625	23	0.534883721	0.090116279
RPH3AL	29	0.725	35	0.813953488	0.088953488
SLC19A3	29	0.725	35	0.813953488	0.088953488
TLX3	29	0.725	35	0.813953488	0.088953488
RAPGEF4	30	0.75	36	0.837209302	0.087209302
NONHSAT037945	30	0.75	36	0.837209302	0.087209302
KCNK10	23	0.575	21	0.488372093	0.086627907
OTX2	23	0.575	21	0.488372093	0.086627907

ARRDC3	36	0.9	35	0.813953488	0.086046512
IQCA1	36	0.9	35	0.813953488	0.086046512
RPE65	36	0.9	35	0.813953488	0.086046512
FGF17	31	0.775	37	0.860465116	0.085465116
ENST00000480646	35	0.875	34	0.790697674	0.084302326
BUB1	32	0.8	38	0.88372093	0.08372093
ZNF586	32	0.8	38	0.88372093	0.08372093
CLCF1	32	0.8	38	0.88372093	0.08372093
SLC41A2	32	0.8	38	0.88372093	0.08372093
ENST00000425514	32	0.8	31	0.720930233	0.079069767
LMX1B	35	0.875	41	0.953488372	0.078488372
LMO3	35	0.875	41	0.953488372	0.078488372
ZNF24	31	0.775	30	0.697674419	0.077325581
ENST00000448964	31	0.775	30	0.697674419	0.077325581
SOCS5	24	0.6	29	0.674418605	0.074418605
ENST00000448204	27	0.675	32	0.744186047	0.069186047
ENST00000563886	25	0.625	24	0.558139535	0.066860465

MIPOL1	29	0.725	34	0.790697674	0.065697674
SNAI1	29	0.725	34	0.790697674	0.065697674
PXT1	37	0.925	37	0.860465116	0.064534884
ENST00000484326	30	0.75	35	0.813953488	0.063953488
CIB2	30	0.75	35	0.813953488	0.063953488
ENST00000554879	30	0.75	35	0.813953488	0.063953488
GPC5	30	0.75	35	0.813953488	0.063953488
NONHSAT016596	30	0.75	35	0.813953488	0.063953488
ENST00000487388	36	0.9	36	0.837209302	0.062790698
TMEM182	36	0.9	36	0.837209302	0.062790698
RNF8	31	0.775	36	0.837209302	0.062209302
NONHSAT022678	31	0.775	36	0.837209302	0.062209302
ARFRP1	31	0.775	36	0.837209302	0.062209302
CPNE6	31	0.775	36	0.837209302	0.062209302
LCN6	31	0.775	36	0.837209302	0.062209302
EYA1	31	0.775	36	0.837209302	0.062209302
NONHSAT041603	35	0.875	35	0.813953488	0.061046512

ENAM	35	0.875	35	0.813953488	0.061046512
NONHSAT099421	32	0.8	37	0.860465116	0.060465116
RRP7A	32	0.8	37	0.860465116	0.060465116
THY1	32	0.8	37	0.860465116	0.060465116
ENST00000485233	32	0.8	37	0.860465116	0.060465116
ENST00000511619	32	0.8	37	0.860465116	0.060465116
ENST00000530646	32	0.8	37	0.860465116	0.060465116
GPR132	34	0.85	34	0.790697674	0.059302326
MRM1	34	0.85	34	0.790697674	0.059302326
ENST00000477213	34	0.85	34	0.790697674	0.059302326
NONHSAT107064	34	0.85	34	0.790697674	0.059302326
GLS2	33	0.825	38	0.88372093	0.05872093
CD53	33	0.825	33	0.76744186	0.05755814
USP46	33	0.825	33	0.76744186	0.05755814
ENST00000460191	34	0.85	39	0.906976744	0.056976744
ENST00000517329	22	0.55	26	0.604651163	0.054651163
DEGS2	31	0.775	31	0.720930233	0.054069767

TXNL4B	23	0.575	27	0.627906977	0.052906977
ADI1	30	0.75	30	0.697674419	0.052325581
ENST00000424657	39	0.975	40	0.930232558	0.044767442
n407559	28	0.7	32	0.744186047	0.044186047
SCN1B	25	0.625	25	0.581395349	0.043604651
TMEM154	25	0.625	25	0.581395349	0.043604651
TMCC3	29	0.725	33	0.76744186	0.04244186
CD300A	29	0.725	33	0.76744186	0.04244186
NONHSAT070986	29	0.725	33	0.76744186	0.04244186
GOLM1	37	0.925	38	0.88372093	0.04127907
EPHB2	30	0.75	34	0.790697674	0.040697674
NONHSAT090384	30	0.75	34	0.790697674	0.040697674
COPS7B	23	0.575	23	0.534883721	0.040116279
ABCF3	36	0.9	37	0.860465116	0.039534884
NEBL	31	0.775	35	0.813953488	0.038953488
TFAP2D	31	0.775	35	0.813953488	0.038953488
ENST00000340779	35	0.875	36	0.837209302	0.037790698

ENST00000489429	35	0.875	36	0.837209302	0.037790698
ENST00000558387	32	0.8	36	0.837209302	0.037209302
ENST00000521423	32	0.8	36	0.837209302	0.037209302
FAM70A	32	0.8	36	0.837209302	0.037209302
ENST00000590093	34	0.85	35	0.813953488	0.036046512
MDGA2	33	0.825	37	0.860465116	0.035465116
CAV2	33	0.825	34	0.790697674	0.034302326
CYP2U1	33	0.825	34	0.790697674	0.034302326
ENST00000459870	32	0.8	33	0.76744186	0.03255814
NONHSAT121756	32	0.8	33	0.76744186	0.03255814
ENST00000478553	35	0.875	39	0.906976744	0.031976744
FEM1A	31	0.775	32	0.744186047	0.030813953
RUNX3	31	0.775	32	0.744186047	0.030813953
ENST00000566547	31	0.775	32	0.744186047	0.030813953
NONHSAT094829	36	0.9	40	0.930232558	0.030232558
ENST00000463783	30	0.75	31	0.720930233	0.029069767
NPFFR2	30	0.75	31	0.720930233	0.029069767

FBXW10	30	0.75	31	0.720930233	0.029069767
B4GALT2	30	0.75	31	0.720930233	0.029069767
NPY1R	37	0.925	41	0.953488372	0.028488372
ENST00000556552	24	0.6	27	0.627906977	0.027906977
IKZF4	29	0.725	30	0.697674419	0.027325581
n405633	29	0.725	30	0.697674419	0.027325581
NONHSAT037627	29	0.725	30	0.697674419	0.027325581
ENST00000587731	25	0.625	28	0.651162791	0.026162791
A4GNT	28	0.7	29	0.674418605	0.025581395
ENST00000588135	27	0.675	30	0.697674419	0.022674419
SPRR3	25	0.625	26	0.604651163	0.020348837
ZNF324	25	0.625	26	0.604651163	0.020348837
ENST00000477626	29	0.725	32	0.744186047	0.019186047
NONHSAT029838	30	0.75	33	0.76744186	0.01744186
SLC25A28	30	0.75	33	0.76744186	0.01744186
MAP3K9	36	0.9	38	0.88372093	0.01627907
PUS7	36	0.9	38	0.88372093	0.01627907

CPT1A	36	0.9	38	0.88372093	0.01627907
RNASE7	31	0.775	34	0.790697674	0.015697674
NONHSAT074002	31	0.775	34	0.790697674	0.015697674
NONHSAT096917	31	0.775	34	0.790697674	0.015697674
SV2B	31	0.775	34	0.790697674	0.015697674
TMC7	31	0.775	34	0.790697674	0.015697674
PLEKHG4B	35	0.875	37	0.860465116	0.014534884
MRPS9	35	0.875	37	0.860465116	0.014534884
MID2	32	0.8	35	0.813953488	0.013953488
GALR1	32	0.8	35	0.813953488	0.013953488
FSTL5	34	0.85	36	0.837209302	0.012790698
WDR77	33	0.825	36	0.837209302	0.012209302
ENST00000488442	33	0.825	36	0.837209302	0.012209302
ENST00000415736	33	0.825	36	0.837209302	0.012209302
ENST00000459720	33	0.825	36	0.837209302	0.012209302
ENST00000508845	33	0.825	35	0.813953488	0.011046512
MEI1	34	0.85	37	0.860465116	0.010465116

ZNF385B	34	0.85	37	0.860465116	0.010465116
NONHSAT096939	21	0.525	23	0.534883721	0.009883721
NONHSAT105370	32	0.8	34	0.790697674	0.009302326
ENST00000463550	32	0.8	34	0.790697674	0.009302326
MFSD4	32	0.8	34	0.790697674	0.009302326
NONHSAT071840	22	0.55	24	0.558139535	0.008139535
ENST00000510590	31	0.775	33	0.76744186	0.00755814
NONHSAT022800	31	0.775	33	0.76744186	0.00755814
MTMR4	23	0.575	25	0.581395349	0.006395349
CXADR	23	0.575	25	0.581395349	0.006395349
IQCG	37	0.925	40	0.930232558	0.005232558
LRRC61	37	0.925	40	0.930232558	0.005232558
ACTN4	29	0.725	31	0.720930233	0.004069767
NONHSAT046696	38	0.95	41	0.953488372	0.003488372
ENST00000526482	39	0.975	42	0.976744186	0.001744186

Table S4 The alignment scores and the thermal stabilities of the free energy of the 46 candidate ceRNA axes related to TGT response

miRNA	lncRNA	mRNA	lncRNA-align_score	lncRNA-energy	Gene-align_score	Gene-energy
hsa-miR-1298-3p	ENST00000461340	PDE9A	150	-19.3	145	-14.34
hsa-miR-185-3p	ENST00000441316	UCKL1	142	-24.67	141	-21.1
hsa-miR-185-3p	ENST00000470741	CASC3	166	-29.86	161	-29.67
hsa-miR-185-3p	ENST00000524768	FAM123B	146	-23.43	141	-18.2
hsa-miR-18b-5p	ENST00000580914	CCDC57	144	-17.61	143	-14.66
hsa-miR-195-5p	ENST00000566103	DMRT2	158	-24.49	144	-12.4
hsa-miR-195-5p	ENST00000567228	DAGLA	146	-17.12	140	-14.5
hsa-miR-195-5p	ENST00000580914	GRAMD1C	148	-10.93	140	-10.29
hsa-miR-21-3p	ENST00000556386	GABRA6	141	-13.56	140	-10.19
hsa-miR-2355-3p	ENST00000441316	UCKL1	151	-22.04	146	-19.26
hsa-miR-2355-3p	ENST00000470741	CASC3	149	-24.67	140	-14.17
hsa-miR-2355-3p	ENST00000470741	CYP4F2	149	-24.67	144	-16.77
hsa-miR-2355-3p	ENST00000487920	NRAS	143	-19.02	140	-15.65
hsa-miR-2355-3p	ENST00000492808	DDTL	156	-21.59	141	-18.17
hsa-miR-2355-3p	ENST00000492808	HSPA2	156	-21.59	141	-17.86

hsa-miR-2355-3p	ENST00000492808	NEK5	156	-21.59	142	-14.36
hsa-miR-2355-3p	ENST00000492808	SARM1	156	-21.59	143	-14.72
hsa-miR-2355-3p	ENST00000567228	DAGLA	153	-23.23	142	-18.29
hsa-miR-2355-3p	ENST00000567228	WDR72	153	-23.23	140	-11.74
hsa-miR-374b-5p	ENST00000505982	PLN	159	-5.42	140	-3.39
hsa-miR-378g	ENST00000471094	DAGLA	164	-23.16	150	-19.6
hsa-miR-378g	ENST00000593706	DMRT2	149	-23.76	147	-18.27
hsa-miR-378g	ENST00000598814	PPP4R4	157	-25.23	148	-24.44
hsa-miR-455-3p	ENST00000600795	PDE9A	151	-18.33	141	-18.21
hsa-miR-4701-5p	ENST00000471094	DAGLA	143	-17.36	140	-13.65
hsa-miR-4701-5p	ENST00000484985	DAGLA	157	-26.21	140	-13.65
hsa-miR-4701-5p	ENST00000484985	ISY1	157	-26.21	151	-17.31
hsa-miR-4701-5p	ENST00000556386	UCKL1	155	-20.26	140	-19.65
hsa-miR-4701-5p	ENST00000567228	DAGLA	143	-23.78	140	-13.65
hsa-miR-4701-5p	ENST00000593706	DMRT2	166	-26.18	142	-12.38
hsa-miR-4701-5p	ENST00000593706	ZC3H10	166	-26.18	144	-24.81
hsa-miR-4720-5p	ENST00000417539	GRM7	146	-18.27	141	-12.94

hsa-miR-497-5p	ENST00000470741	CASC3	143	-27.22	142	-19.87
hsa-miR-497-5p	ENST00000556386	AMOT	151	-19.94	140	-11.69
hsa-miR-497-5p	ENST00000566103	DMRT2	156	-24.18	146	-18.2
hsa-miR-497-5p	ENST00000580914	CCDC57	150	-16.75	144	-16.18
hsa-miR-497-5p	ENST00000580914	GRAMD1C	150	-16.75	147	-12.48
hsa-miR-505-3p	ENST00000566103	DMRT2	146	-18.34	144	-9.28
hsa-miR-550a-5p	ENST00000569390	ARHGAP28	149	-21.36	142	-17.42
hsa-miR-654-5p	ENST00000494760	C1QC	150	-25.87	142	-18.76
hsa-miR-654-5p	ENST00000549080	SELV	150	-24.99	143	-19.16
hsa-miR-654-5p	ENST00000585828	C1QC	151	-19.75	142	-18.76
hsa-miR-7-1-3p	ENST00000524688	NRAS	143	-12.7	141	-5.25
hsa-miR-7154-5p	ENST00000417539	GRM7	142	-15.96	140	-13.13
hsa-miR-744-3p	ENST00000556386	SLC35E3	145	-14.93	140	-10.2
hsa-miR-941	ENST00000471094	DAGLA	149	-28.75	141	-25.79
